# Supplementary material for: Actigraphy assessment of motor activity and sleep in patients with alcohol withdrawal syndrome and the effects of intranasal oxytocin
Source: PLoS One. 2020 Feb 13;15(2):e0228700. doi: 10.1371/journal.pone.0228700 (PMC7018062; doi:10.1371/journal.pone.0228700)
Supplement: S2 Table — Panel A. In 20 patients receiving placebo. Panel B. In 20 patients receiving oxytocin. (DOCX) [file pone.0228700.s003.docx]

**S2 Table.** **Correlation between clinical variables related to alcohol intake and withdrawal, and actigraphy recordings of sleep variables in patients with alcohol use disorder in the second night during detoxification from alcohol.**

|  | **Mean/min** | | **SD/min in % of mean** | | **RMSSD/min in % of mean** | | **RMSSD/SD** | |
| --- | --- | --- | --- | --- | --- | --- | --- | --- |
|  | **r** | **P value** | **r** | **P value** | **r** | **P value** | **r** | **P value** |
| Self-reported daily alcohol intake last 14 days (standard alcohol units^1^), mean ± SD | 0.05 | 0.84 | 0.23 | 0.32 | 0.43 | 0.060 | **0.54** | **0.014** |
| Phosphatidyl-ethanol blood concentration, (µmol/L), mean ± SD^2^ | -0.02 | 0.94 | -0.03 | 0.90 | 0.12 | 0.64 | 0.21 | 0.38 |
| Total CIWA-Ar score | -0.18 | 0.45 | 0.30 | 0.20 | 0.34 | 0.14 | 0.30 | 0.20 |
| CIWA-Ar score,  tremor | 0.42 | 0.069 | -0.36 | 0.13 | -0.22 | 0.34 | 0.09 | 0.70 |
| CIWA-Ar score,  agitation | 0.07 | 0.76 | 0.08 | 0.73 | 0.04 | 0.86 | -0.08 | 0.73 |
| CIWA-Ar score,  anxiety | 0.07 | 0.76 | 0.05 | 0.84 | 0.12 | 0.62 | 0.20 | 0.40 |
| Oxazepam  dose | -0.01 | 0.83 | 0.18 | 0.45 | 0.20 | 0.40 | 0.14 | 0.48 |

**Panel A. In 20 patients receiving placebo.**

|  | **Sleep duration**  **(min)** | | **Sleep efficiency^2^ (%)** | | **Total sleep time^3^ (min)** | | **Total sleep time (%)** | |
| --- | --- | --- | --- | --- | --- | --- | --- | --- |
|  | **r** | **P value** | **r** | **P value** | **r** | **P value** | **r** | **P value** |
| Self-reported daily alcohol intake last 14 days (standard alcohol units^1^), mean ± SD | 0.09 | 0.70 | 0.29 | 0.23 | 0.13 | 0.60 | 0.24 | 0.32 |
| Phosphatidylethanol blood concentration, (µmol/L), mean ± SD | -0.06 | 0.82 | 0.11 | 0.65 | -0.11 | 0.67 | -0.09 | 0.71 |
| Total CIWA-Ar score | -0.09 | 0.73 | 0.16 | 0.52 | -0.01 | 0.96 | 0.20 | 0.40 |
| Oxazepam dose | -0.02 | 0.93 | 0.24 | 0.33 | 0.05 | 0.85 | 0.25 | 0.31 |
| Self-reported sleep | **0.51** | **0.043** | 0.36 | 0.17 | **0.50** | **0.047** | 0.16 | 0.55 |

CIWA-Ar = Clinical Institute of Withdrawal Assessment – Alcohol revised

SD = standard deviation

Statistically significant correlations are shown in bold

^1^ One standard alcohol unit corresponds to 12.8 g ethanol

^2^ Ratio between total sleep time and total duration of time in bed

^3^ Duration of sleep during the major sleep period in the evening/night

**Panel B.** **In 20 patients receiving oxytocin.**

|  | **Sleep duration**  **(min)** | | **Sleep efficiency^2^ (%)** | | **Total sleep time^3^ (min)** | | **Total sleep time (%)** | |
| --- | --- | --- | --- | --- | --- | --- | --- | --- |
|  | **r** | **P value** | **r** | **P value** | **r** | **P value** | **r** | **P value** |
| Self-reported daily alcohol intake last 14 days (standard alcohol units^1^), mean ± SD | **0.48** | **0.037** | 0.13 | 0.59 | 0.38 | 0.11 | -0.42 | 0.071 |
| Phosphatidylethanol blood concentration, (µmol/L), mean ± SD | 0.001 | 1.00 | -0.01 | 0.97 | -0.04 | 0.86 | -0.09 | 0.73 |
| Total CIWA-Ar score | 0.28 | 0.25 | 0.30 | 0.22 | 0.26 | 0.29 | -0.06 | 0.80 |
| Oxazepam dose | 0.15 | 0.54 | 0.20 | 0.41 | 0.15 | 0.54 | 0.01 | 0.98 |
| Self-reported sleep | 0.29 | 0.35 | **0.60** | **0.029** | 0.31 | 0.30 | 0.23 | 0.44 |

CIWA-Ar = Clinical Institute of Withdrawal Assessment – Alcohol revised

SD = standard deviation

Statistically significant correlations are shown in bold

^1^ One standard alcohol unit corresponds to 12.8 g ethanol

^2^ Ratio between total sleep time and total duration of time in bed

^3^ Duration of sleep during the major sleep period in the evening/night
