# Supplementary material for: Actigraphy assessment of motor activity and sleep in patients with alcohol withdrawal syndrome and the effects of intranasal oxytocin
Source: PLoS One. 2020 Feb 13;15(2):e0228700. doi: 10.1371/journal.pone.0228700 (PMC7018062; doi:10.1371/journal.pone.0228700)
Supplement: S3 Table — (DOCX) [file pone.0228700.s004.docx]

**S3 Table. Number of subjects with alcohol use disorder overestimating, accurately estimating and underestimating their sleep duration in relation to actigraphy-recorded sleep during the first and second nights of acute detoxification.**

|  | **First night (n=29)** | **Second night (n=28)** |
| --- | --- | --- |
| **Total group** | | |
| Overestimating | 8 (28 %) | 13 (46 %) |
| Accurately estimating ^1^ | 10 (35 %) | 5 (18 %) |
| Underestimating | 11 (38 %) | 10 (36 %) |
| **Oxytocin group** | | |
| Overestimating | 5 (38 %) | 6 (46 %) |
| Accurately estimating ^1^ | 5 (38 %) | 3 (23 %) |
| Underestimating | 3 (23 %) | 4 (30 %) |
| **Placebo group** | | |
| Overestimating | 3 (19 %) | 7 (47 %) |
| Accurately estimating ^1^ | 5 (31 %) | 2 (13 %) |
| Underestimating | 8 (50 %) | 7 (40 %) |

^1^ Defined as a self-reported sleep duration within ± 10 % of the actigraphy-recorded sleep duration.
