# Supplementary material for: Actigraphy assessment of motor activity and sleep in patients with alcohol withdrawal syndrome and the effects of intranasal oxytocin
Source: PLoS One. 2020 Feb 13;15(2):e0228700. doi: 10.1371/journal.pone.0228700 (PMC7018062; doi:10.1371/journal.pone.0228700)
Supplement: S2 Protocol — (DOCX) [file pone.0228700.s006.docx]

ENDRINGsmelding TIL REK 09.08.16

Prosjekttittel:

**ACUTE AND LONG-TERM EFFECTS OF INTRANASAL OXYTOCIN IN ALCOHOL WITHDRAWAL AND DEPENDENCE: A PROSPECTIVE RANDOMIZED PARALLEL GROUP PLACEBO-CONTROLLED TRIAL**

Protocol Identification Number:140682

EudraCTNumber: 2015-004463-37

Personer som inkluderes i studien er voksne pasienter som samtykker til deltakelse i studien før inklusjon. Oxytocin nesespray; «Syntocinon», Sigma-Tau er et godkjent legemiddel i Norge. En lignende studie med samme doser intranasal oxytocin er gjennomført på pasienter innlagt for alkoholavrusning (Pedersen et al., 2013). Ingen kjente bivirkninger er funnet ved gjennomgang av studier med kortvarig bruk av oxytocin i doser 18-40 IU (Macdonald et al., 2011).

Prosjektet ble godkjent av REK Midt på møte 05.02.2016, saksnummer **2016/45/REK midt**

| Acute and long-term effects of intranasal oxytocin in alcohol withdrawal and  dependence: A prospective randomized PARALLEL GROUP placebo-controlled trial |
| --- |

**Protocol Identification Number:** 140682

**EudraCT Number:** 2015-004463-37

| **SPONSOR:** | **Trond Jacobsen, Head of Clinic of Laboratory Medicine**  **St. Olav’s University Hospital**  Address St. Olav’s University Hospital, Clinic of Laboratory Medicine, PO box 3250 Sluppen, 7006 Trondheim, Norway  Tel : 06800  E-mail: Trond.Jacobsen@stolav.no |
| --- | --- |

| **PRINCIPAL INVESTIGATOR (PI):** | **Olav Spigset, MD, PhD, Professor of clinical pharmacology and senior consultant, NTNU and St.Olav’s University Hospital**  Address St. Olav’s University Hospital, Clinic of Laboratory Medicine, Department of Clinical Pharmacology, PO box 3250 Sluppen, 7006 Trondheim, Norway  Tel : 06800  E-mail: olav.spigset@legemidler.no |
| --- | --- |

| **PROTOCOL VERSION NO. 3 09.08.2016** |
| --- |
|  |

# CONTACT details

| **Sponsor:** | **Trond Jacobsen, Head of Clinic of Laboratory Medicine**  **St. Olav’s University Hospital**  Address: St. Olav’s University Hospital, Clinic of Laboratory Medicine, PO box 3250 Sluppen, N-7006 Trondheim, Norway  Tel : 06800  E-mail: Trond.Jacobsen@stolav.no |
| --- | --- |
| **Principal investigator:** | **Olav Spigset, MD, PhD, Professor of clinical pharmacology and senior consultant, NTNU and St.Olav’s University Hospital**  Address: St. Olav’s University Hospital, Clinic of Laboratory Medicine, Department of Clinical Pharmacology, PO box 3250 Sluppen, N-7006 Trondheim, Norway  Tel : 06800  E-mail: olav.spigset@legemidler.no |
| **Participating Departments:** | **Lade Addiction Treatment Center**  Address: Lade Allé 86, N-7041 Trondheim, Norway  Tel : 73848500  E-mail: post@ladebs.no |
| **Monitor:** | **Unit for Applied Clinical Research**  Address. NTNU, AKF, Faculty of Medicine, PO box N-7491, Trondheim, Norway  Tel : 72571109 |

# Signature page

| Title | Acute and long-term effects of intranasal oxytocin in alcohol withdrawal and dependence: A prospective randomized parallel group placebo-controlled trial |
| --- | --- |
| Protocol ID no: | 140682 |
| EudraCT no: | 2015-004463-37 |

***I hereby declare that I will conduct the study in compliance with the Protocol, ICH GCP and the applicable regulatory requirements:***

| **Name** | **Title** | **Role** | **Signature** | **Date** |
| --- | --- | --- | --- | --- |
| Olav Spigset | M.D., Professor of Clinical Pharmacology, Norwegian University of Science and Technology, Faculty of Medicine; Senior Medical Consultant, .St.Olav’s University Hospital | Principal Investigator | _______________ |  |
| Rolf W. Gråwe | Clinical Psychologist, PhD, Professor of Addiction Medicine, NTNU, Head of R & D, Clinic for Substance Use and Addiction Medicine, St.Olav’s University Hospital | Co-investigator | ________________ |  |
| Trond Aamo | M.D., Head of Clinic of Laboratory Medicine, Department of Clinical Pharmacology, St. Olav’s University Hospital, Physician at Central Norway Regional Health Authority, Lade Addiction Treatment Center | Co-investigator | ________________ |  |
| Trond Jacobsen | M.D., Head of Clinic of Laboratory Medicine, St. Olav’s University Hospital | Sponsor | ________________ |  |
| Øyvind Salvesen | Assistant Professor, statistician, Norwegian University of Science and Technology, Unit for Applied Research, Faculty of Medicine | Statistician | ________________ |  |
| Hanna Gunnestad | Physician, Central Norway Regional Health Authority, Lade Addiction Treatment Center | Co-investigator | ________________ |  |
| Mari Tvervaag Solbakken | Physician, Central Norway Regional Health Authority, Lade Addiction Treatment Center | Co-investigator | ________________ |  |
| Katrine Melby | Physician, Central Norway Regional Health Authority, Lade Addiction Treatment Center | PhD-candidate | ________________ |  |

# PROTOCOL SYNOPSIS

| Acute and long-term effects of intranasal oxytocin in alcohol withdrawal and dependence: A prospective randomized parallel group placebo-controlled trial |
| --- |

| Sponsor | Trond Jacobsen, St. Olav’s University Hospital |
| --- | --- |
| Phase and study type | Phase III |
| Investigational Medical Product (IMP) (including active comparator and placebo) : | Syntocinon nasal spray and placebo |
| Center: | Lade Addiction Treatment Center, Central Norway Regional Health Authority |
| Study Period: | Estimated date of first patient enrolled: 01.10.2016  Anticipated recruitment period: 12 months  Estimated date of last patient completed: 01.10.2017 |
| Treatment Duration: | 3 days in-patient treatment, followed by 4 weeks outpatient treatment and phone interview at 8 weeks. |
| Follow-up: | 4 weeks follow-up during outpatient treatment, phone interview at 8 weeks. |
| Objectives | The primary aim of the study is to test whether daily intranasal administration of oxytocin is more effective than placebo in decreasing the oxazepam dosages required to control withdrawal symptoms during a 3-day inpatient program of medical detoxification. The secondary aims are: 1) test whether daily intranasal administration of oxytocin is more effective than placebo in decreasing CIWA-Ar scores during the detoxification period; 2) test the effects on therapist- and self-reported withdrawal symptoms, dysphoria and Actigraph-assessed akathisia and sleep during the detoxification period; and 3) test whether daily intranasal self-administration of oxytocin is more effective than placebo on alcohol use measures (time to ‘relapse’ and amount, cravings), quality of sleep and mood states during a subsequent 4 week out-patient follow-up period. |
| Endpoints: | Primary endpoint: Phase 1: Milligrams of benzodiazepines (oxazepam) used to complete 3 days of detoxification and Actigraph-assessed akathisia and sleep. Phase 2: Time to ‘relapse’ and amount of alcohol intake during 4 weeks of subsequent outpatient treatment.  Secondary endpoint: Phase 1: CIWA-Ar-score, effects on therapist- and self-reported withdrawal symptoms, dysphoria, effects on mood, craving, sleep. |
| Study Design: | Double blind, prospective randomized parallel group placebo-controlled trial. |
| Main Inclusion Criteria: | 1) At least one prior episode 2 days or longer in duration during which the subject experienced withdrawal symptoms that caused significant incapacitation (e.g., inability to work or do normal activities) OR at least one prior inpatient or outpatient medical detoxification during which the subject exhibited withdrawal symptoms of sufficient magnitude that sedative-hypnotic or anticonvulsant medication was required at least once on 2 consecutive days after cessation of or reduction in the use of alcohol following 2 weeks or more of heavy daily consumption; 2) average consumption of 8-30 standard drinks per day for at least 2 weeks prior to enrollment in the study; 3) age 18-65; 4) consenting to participate in the study; 5) have residency in Trøndelag County after discharge |
| Main Exclusion Criteria | 1) chronic treatment with sedative-hypnotic medications such as benzodiazepines or z-hypnotica; 2) dependence on substances other than alcohol, nicotine or caffeine; 3) inadequately treated, unstable and/or compromising medical or psychiatric conditions; 4) low body weight (BMI < 17) or history of anorexia nervosa or bulimia in the past 2 years; 5) pregnancy; parturition or breast-feeding in the past 6 months; 6) inability to read well enough to complete study questionnaires determined by whether the prospective subject can read the consent form without help and correctly answer basic questions about information in the consent form; 7) no alcohol in the blood and > 15 h since last intake of alcohol; 8) prior inclusion and participation in the same study.  Eligible patients will be identified by the intake team and receive information about the trial together with other standard pre-hospitalization information one week before admittance. Patients admitted on short notice will also be eligible for the study. The randomization will be administrated electronically by the Unit of Applied Clinical Research at the Norwegian University of Science and Technology. |
| Sample Size: | 40 patients |
| Efficacy Assessments: | Milligrams of benzodiazepines (oxazepam) needed to complete 3 days of detoxification. |
| Safety Assessments: | Clinical examination, biochemistry/hematology/toxicology and medical history on admittance to decide if patient meet inclution criterias. Negative pregnancytest. CIWA-Ar with vital signs. |

# TABLE OF CONTENTS

[CONTACT details 3](#_Toc452967349)

[Signature page 4](#_Toc452967350)

[PROTOCOL SYNOPSIS 5](#_Toc452967351)

[TABLE OF CONTENTS 7](#_Toc452967352)

[List of Abbreviations and Definitions of Terms 10](#_Toc452967353)

[1 introduction 10](#_Toc452967354)

[1.1 Background – Alcohol withdrawal syndrome 10](#_Toc452967355)

[1.2 Background - Therapeutic Information 11](#_Toc452967356)

[1.3 Pre-Clinical & Clinical Experience with Oxytocin 11](#_Toc452967357)

[1.4 Rationale for the Study and Purpose 11](#_Toc452967358)

[2 STUDY OBJECTIVES and related endpoints 12](#_Toc452967359)

[2.1 Primary Endpoint 12](#_Toc452967360)

[2.2 Secondary Endpoints 12](#_Toc452967361)

[3 Overall STUDY Design 12](#_Toc452967362)

[4 STUDY POPULATION 13](#_Toc452967363)

[4.1 Selection of Study Population 13](#_Toc452967364)

[4.2 Number of Patients 13](#_Toc452967365)

[4.3 Inclusion Criteria 13](#_Toc452967366)

[4.4 Exclusion Criteria 14](#_Toc452967367)

[5 TREATMENT 14](#_Toc452967368)

[5.1 Drug Identity, Supply and Storage 14](#_Toc452967369)

[5.2 Dosage and Drug Administration 16](#_Toc452967373)

[5.3 Duration of Therapy 16](#_Toc452967374)

[5.4 Premedication and Monitoring 16](#_Toc452967375)

[5.5 Concomitant Medication 17](#_Toc452967376)

[5.6 Subject Compliance 17](#_Toc452967377)

[5.7 Drug Accountability 17](#_Toc452967378)

[5.8 Drug Labeling 17](#_Toc452967379)

[5.9 Subject Numbering 18](#_Toc452967380)

[6 STUDY procedures 18](#_Toc452967381)

[6.1 Flow Chart 18](#_Toc452967382)

[6.2 On arrival 19](#_Toc452967383)

[6.2.1 Treatment Start, D1 20](#_Toc452967384)

[6.2.2 During Treatment, D1-D3 20](#_Toc452967385)

[6.2.3 End of Treatment and End of Study Visit, D 30 20](#_Toc452967386)

[6.2.4 Withdrawal Visit 20](#_Toc452967387)

[6.3 Criteria for Patient Discontinuation 20](#_Toc452967388)

[6.4 Procedures for Discontinuation 21](#_Toc452967389)

[6.4.1 Patient Discontinuation 21](#_Toc452967390)

[6.4.2 Trial Discontinuation 21](#_Toc452967391)

[6.5 Laboratory Tests 21](#_Toc452967392)

[7 assessments 21](#_Toc452967393)

[7.1 Assessment of Efficacy Response 21](#_Toc452967394)

[7.2 Safety and Tolerability Assessments 21](#_Toc452967395)

[7.3 Other Assessments 22](#_Toc452967396)

[8 Safety MONItoring and reporting 23](#_Toc452967397)

[8.1 Definitions 23](#_Toc452967398)

[8.1.1 Adverse Event (AE) 23](#_Toc452967399)

[8.1.2 Serious Adverse Event (SAE) 23](#_Toc452967400)

[8.1.3 Suspected Unexpected Serious Adverse Reaction (SUSAR) 24](#_Toc452967401)

[8.2 Expected Adverse Events 24](#_Toc452967402)

[8.3 Time Period for Reporting AE and SAE 24](#_Toc452967403)

[8.4 Recording of Adverse Events 24](#_Toc452967404)

[8.5 Reporting Procedure 25](#_Toc452967405)

[8.5.1 AEs and SAEs 25](#_Toc452967406)

[8.5.2 SUSARs 25](#_Toc452967407)

[8.5.3 Annual Safety Report 26](#_Toc452967408)

[8.5.4 Clinical Study Report 26](#_Toc452967409)

[8.6 Procedures in Case of Emergency 26](#_Toc452967410)

[9 Data management and monitoring 26](#_Toc452967411)

[9.1 Case Report Forms (CRFs) 26](#_Toc452967412)

[9.2 Source Data 26](#_Toc452967413)

[9.3 Study Monitoring 27](#_Toc452967414)

[9.4 Confidentiality 27](#_Toc452967415)

[9.5 Database management 27](#_Toc452967416)

[10 Statistical methods and data analysis 28](#_Toc452967417)

[10.1 Determination of Sample Size 28](#_Toc452967418)

[10.2 Randomization 28](#_Toc452967419)

[10.2.1 Allocation- procedure to randomize a patient 28](#_Toc452967420)

[10.2.2 Blinding and emergency unblinding 28](#_Toc452967421)

[10.3 Population for Analysis 28](#_Toc452967422)

[10.4 Statistical Analysis 28](#_Toc452967423)

[11 STUDY MANAGEMENT 29](#_Toc452967424)

[11.1 Investigator Delegation Procedure 29](#_Toc452967425)

[11.2 Protocol Adherence 29](#_Toc452967426)

[11.3 Study Amendments 29](#_Toc452967427)

[11.4 Audit and Inspections 29](#_Toc452967428)

[12 Ethical and regulatory requirements 29](#_Toc452967429)

[12.1 Ethics Committee Approval 30](#_Toc452967430)

[12.2 Other Regulatory Approvals 30](#_Toc452967431)

[12.3 Informed Consent Procedure 30](#_Toc452967432)

[12.4 Subject Identification 30](#_Toc452967433)

[13 Trial sponsorship and financing 30](#_Toc452967434)

[14 Trial insurance 30](#_Toc452967435)

[15 Publication policy 30](#_Toc452967436)

[16 REFERENCES 31](#_Toc452967437)

[17 LIST OF APPENDICES 32](#_Toc452967438)

[Appendix A – Specifiations of nasal spray pump and spray quality 33](#_Toc452967439)

[Appendix B – CIWA-Ar guidelines 36](#_Toc452967441)

[Appendix C – Placebo decleration list 38](#_Toc452967442)

[Appendix D – example of labelling 39](#_Toc452967443)

[Appendix E – Trial insurance 41](#_Toc452967444)

# List of Abbreviations and Definitions of Terms

| **Abbreviation or special term** | **Explanation** |
| --- | --- |
| ACE | Alcohol Craving Experience Questionnaire |
| AE | Adverse Event |
| ACVAS | Alcohol Craving Visual Analog Scale |
| AWS | Alcohol Withdrawal Syndrome |
| AWSC | Alcohol Withdrawal Syndrome Checklist |
| BAI | Beck Anxiety Inventory |
| BDI | Beck Depression Inventori |
| CIWA-Ar | Clinical Institute Withdrawal Assessment-Alcohol revised |
| CNS | Central Nervous System |
| CRF | Case Report Form (electronic/paper) |
| CSA | Clinical Study Agreement |
| CTC | Common Toxicity Criteria |
| CTCAE | Common Terminology Criteria for Adverse Event |
| DAE | Discontinuation due to Adverse Event |
| EC | Ethics Committee, synonymous to Institutional Review Board (IRB) and Independent Ethics Committee (IEC) |
| GABA | gamma-Aminobutyric acid |
| GCP | Good Clinical Practice |
| IB | Investigator’s Brochure |
| ICF | Informed Consent Form |
| ICH | International Conference on Harmonization |
| IMP | Investigational Medicinal Product (includes active comparator and placebo) |
| IND | Investigational New Drug |
| MDMA | 3,4-methylenedioxy-methamphetamine |
| NOMA | Norwegian Medical Agency |
| PAN | Psychotropic Analgesic Nitrous Oxide |
| POMS | Profile of Moods States |
| SAE | Serious Adverse Event |
| SCL-10 | Symptom check list -10 |
| SD | Stable Disease |
| SOP | Standard Operating Procedure |

# introduction

## Background – Alcohol withdrawal syndrome

Alcohol use disorder represents a serious health problem and alcohol dependence is associated with physiological (tolerance and withdrawal) and behavioural (reduced drinking control) symptoms and has interpersonal, social and legal consequences. Alcohol withdrawal syndrome (AWS) is a cluster of symptoms that frequently occur in alcohol-dependent persons and that develops after cessation or reduced intake of alcohol ^1^. Syndromatic symptoms as autonomic and central hyperactivity (sweating, tachycardia, tremor, insomnia, agitation, psychotic symptoms, seizures, anxiety), are mainly due to the GABA-suppressive and glutamate-activating effects of alcohol leading to central nervous system (CNS) hyperactivity in the absence of alcohol. The symptoms may vary from mild to serious and can appear up to 72 hours after last intake (included delirium tremens).Advances in neurobiology and neurochemistry have led to the use of pharmaceuticals in the treatment of alcohol dependence and AWS.

## Background - Therapeutic Information

Common treatments of AWS are benzodiazepines, anticonvulsants, baclofen, gammahydroxy butyrate and psychotropic analgesic nitrous oxide (PAN). Of these only benzodiazepines have shown to perform significantly better than placebo in reducing AWS ^2^. Benzodiazepines have, however, a high potential to be addictive, increase morbidity and mortality, may have depressive respiratory effects when administered in combination with alcohol use, and have limited use in outpatient treatment. At Lade Addiction Treatment Center in Trondheim, oxazepam is the preferred drug to inpatients receiving alcohol detoxification. Doses are given based on withdrawal symptoms measured by Clinical Institute Withdrawal Assessment-Alcohol revised ^3^. Treatment is also supplemented with anticonvulsants and vitamin B following guidelines for dosages.

## Pre-Clinical & Clinical Experience with Oxytocin

Oxytocin is a neuropeptide with synthesis both in the central nervous system and in peripheral tissues such as the uterus, ovaries, testis, vascular endothelium cells and the heart. It is centrally produced by the paraventricular nucleus and the supraoptic nucleus of the hypothalamus, and stored and released from the posterior pituitary into the blood stream (when for example triggered by nipple or vagocervical stimulation). Research suggests that oxytocin has anxiolytic effects and that there is a reciprocal association between positive social stimulation (social bonding) and oxytocin levels^4^. Its anxiety reducing effect has been explored in many studies in the last years as a potential drug to use for patients suffering from psychiatric disorders ^5^. In particular, oxytocin has been suggested to facilitate social reward mechanisms and to support consecutively direct rewarding effects of drugs of abuse ^6^. Brain imaging studies report that MDMA (“ecstasy”) intake, which is associated with central oxytocin release ^7^, alleviates amygdala responses toward unpleasant social stimuli while increasing it toward pleasant stimuli ^8^. Several human studies suggest that intranasal oxytocin increases its concentration in the brain ^9^;^10^;^11^ and enhance social bonding measures^12^, and one study have shown that multiple daily doses reduce psychotic and dissocial schizophrenic symptoms^13^. There are not reported any adverse effects of such oxytocin administration. A review article on intranasal oxytocin in human research showed no detectable subjective changes in recipients, no reliable side effects and was not associated with adverse outcomes in doses of 18-40 IU for short time use^14^. Many animal studies have shown that oxytocin enhance neuroadaptation to alcohol ^15^ and central or peripheral oxytocin reduce alcohol tolerance to the hypothermic, myorelaxant, akinetic and hypnotic effects of alcohol in mice^16^. However, the mechanisms by which oxytocin inhibit tolerance is yet unknown. A small randomized placebo-controlled trial (n=11) of intranasal oxytocin against alcohol withdrawal has recently been conducted ^17^. The authors found that intranasal oxytocin blocked withdrawal symptoms of alcohol in humans as assessed by reduced administration of lorazepam during detoxification, reduced withdrawal symptoms (as measured by the Alcohol Withdrawal Symptom Checklist/AWSC, and the Clinical Institute Withdrawal Assessment for Alcohol score/CIWA) alcohol cravings (as measured by the Alcohol Craving Visual Analog Scale/ACVAS), and tension-anxiety (as measured by the Profile of Mood States/POMS scale). This is the first human indication that alleviated alcohol effects of oxytocin, and the results are consistent with those seen in animal studies.

## Rationale for the Study and Purpose

The study will be conducted at Lade Addiction Treatment Center, Norway. 40 consecutively admitted in-patients undergoing medical detoxification from alcohol at Lade Addiction Treatment Center will be included in the study. In 2014, 150 patients with alcohol dependency as their primary diagnosis were admitted electively to the detoxification unit. If the enrollment of the target number of inclusions exceeds 12 months, the study will seek to include patients from the Clinic of Substance Use and Addiction Medicine at St. Olav’s Hospital.

Oxytocin is neuropeptide hormone which, to our knowledge, is not associated with severe adverse effects. It is a world-wide used, approved, licensed and recommended medicine indicated for the initiation or improvement of uterine contractions in order to achieve vaginal delivery for fetal or maternal reasons. Oxytocin nasal spray is also commonly used by mothers when breastfeeding in the therapy and prophylaxis of mastitis. The potential benefits of the drug as an alternative to benzodiazepine against alcohol withdrawal symptoms, and the benefits of the study are considered greater than the health risks.

In a double blind, prospective randomized parallel group placebo-controlled trial patients will be assigned to receive either oxytocin or placebo which will be administered as a nasal spray twice daily during 3 days of inpatient detoxification, and as a self-administrated spray during a 4-week follow-up period. One arm will receive oxytocin nasal spray, the other arm placebo spray. Patients are randomized in a 1:1 ratio to oxytocin or placebo. The oxytocin will be administered as Syntocinon nasal spray. Syntocinon contains synthetic oxytocin for intranasal use containing 6.7 μg per dose. 1 IU varies between 1.7 μg and 2.2 μg. With 1 IU = 1.7 μg, 1 μg = 0,588 IU, which makes 6.7 μg = 4.34 IU, for practical use 4 IU per dose. We are planning to use 6 insufflations and in total 24 IU given twice daily. This is the same dose of oxytocin as given in a pilot study on human subjects, in which no adverse side effects were reported. The oxytocin dose used by breast-feeding mothers is 6.7 μg.

The placebo spray contains the same ingredients as Syntocinon, except oxytocin.

# STUDY OBJECTIVES and related endpoints

## Primary Endpoint

| Phase 1: Milligrams of benzodiazepines (oxazepam) used to complete 3 days of detoxification and Actigraph-assessed akathisia and sleep.  Phase 2: Time to ‘relapse’ and amount of alcohol intake during 4 weeks of subsequent outpatient treatment. |
| --- |

## Secondary Endpoints

Phase 1: Test whether daily intranasal administration of oxytocin is more effective than placebo in decreasing CIWA-Ar scores, effects on therapist- and self-reported withdrawal symptoms, effects on mood, craving, sleep.

# Overall STUDY Design

The study is a phase III.

Double blind, prospective randomized parallel group placebo-controlled trial.

4 weeks outpatient treatment

Day 1-3: In-patient

treatment

On admission

____________________________________________________________________________________________
Main assessments Study entry Discharge Follow-up
 D0 D2 D3 D30 + D60

Arm 2:Placebo

Arm 1: Oxytocin

| Study Period | Estimated date of first patient enrolled: 01.10.2016  Anticipated recruitment period: 12 months  Estimated date of last patient completed: 01.10.2017 |
| --- | --- |
| Treatment Duration: | 3 days in-patient treatment, followed by 4 weeks outpatient treatment and a phone interview at 8 weeks. |
| Follow-up: | Check-point at 4 weeks during 4-week outpatient treatment, phone interview at 8 weeks |

# STUDY POPULATION

## Selection of Study Population

The study will be conducted at Lade Addiction Treatment Center, St. Olavs University Hospital, Norway. 40 consecutively admitted in-patients undergoing medical detoxification from alcohol at Lade Addiction Treatment Center will be included in the study.Eligible patients will be identified by the intake team and receive information about the trial together with other standard pre-hospitalization information one week before admittance. Patients admitted on short notice will also be eligible for the study. The randomization will be administrated electronically by the Unit of Applied Clinical Research at the Norwegian University of Science and Technology.

## Number of Patients

40 patients will be included in this trial.

## Inclusion Criteria

All of the following conditions must apply to the prospective patient at screening prior to receiving study agent (e.g.):

- At least one prior episode 2 days or longer in duration during which the subject experienced withdrawal symptoms that caused significant incapacitation (e.g., inability to work or do normal activities) OR at least one prior inpatient or outpatient medical detoxification during which the subject exhibited withdrawal symptoms of sufficient magnitude that sedative-hypnotic or anticonvulsant medication was required at least once on 2 consecutive days after cessation of or reduction in the use of alcohol following 2 weeks or more of heavy daily consumption
- Average consumption of 8-30 standard drinks per day for at least 2 weeks prior to enrollment in the study
- Age 18-65
- Have residency inTrøndelag County after discharge
- Signed informed consent and expected cooperation of the patients for the treatment and follow up must be obtained and documented according to ICH GCP, and national/local regulations.

## Exclusion Criteria

Patients will be excluded from the study if they meet any of the following criteria:

- No alcohol in the blood and more than 15 h since last intake of alcohol at admission
- Adverse reactions to some of the ingredients of Syntoinon nasal spray, e.g. allergic reactions to methyl- and propylparahydroksybenzoat (E 216, E 218) or hereditary fructose intolerance
- Chronic treatment with sedative-hypnotic medications such as benzodiazepines or z-hypnotica
- Dependence on substances other than alcohol, nicotine or caffeine
- Inadequately treated, unstable and/or compromising medical or psychiatric conditions
- Low body weight (BMI < 17) or history of anorexia nervosa or bulimia in the past 2 years
- Pregnancy (females at fertile age will undergo pregnancy testing upon admission); parturition or breast-feeding in the past 6 months; females at fertile age not using oral contraceptives or other hormonal conctaceptive products (patch, i.m. depot injections, ring, implant, IUD).
- Inability to read well enough or understand to complete study questionnaires determined by whether the prospective subject can read the consent form without help and correctly answer basic questions about information in the consent form
- Concomitant participation in another medical clinical trial
- Prior inclusion and participation in the same study.
- Any reason why, in the opinion of the investigator, the patient should not participate.

# TREATMENT

For this study Syntocinon nasal spray is defined as Investigational Medicinal Product (IMP). IMP includes also placebo.

## Drug Identity, Supply and Storage

Syntocinon nasal spray 5 ml is decanted into 10 ml amber glass intranasal spray bottles. The same glass bottles will be used for placebo. Unopened (decanted Syntocinon or manufactured placebo) nasal sprays are to be stored chilled, after opening to be stored in room-temperature. A bottle should be used within 1 month after opening.

**IMP and placebo**

The company Sanivo Pharma AS, previously known as Farmaholding (www.farmaholding.no) will decanter Syntocinon into 10 ml amber glass bottles. The company will also manufacture placebo in identical bottles. The study drug will be decanted to new nasal spray containers, following a Class D regimen. Since the production of placebo and the production of Syntocinon take place in two different locations, testing of absence of oxytocin is considered to be unnecessary.

**The glass bottle and nasal spray pump**

The nasal spray bottles are 10 ml snap-on vials manufactured by Gerresheimer Chalon, France ([www.Gerresheimer.com](http://www.Gerresheimer.com)). They are used for nasal sprays manufactured by GSK, Merck, Sanofi, Abbott and others. The nasal spray pumps are manufactured by Aeropump ([www.aeropump.de](http://www.aeropump.de)). They are currently in use for the following drugs internationally: Nasivin (Merck), Olynth (Pfizer), Iliadin (Merck), Mar Plus (Stada) and Prevalin (Teva). In Sweden they are also used for Nozoil (Trimb) and Renässans (Maxmedica). Both glass bottles and nasal spray pump are provided by Wirth Emballage ([www.wirth.se](http://www.wirth.se)).

**Testing of pH and microbiological testing**

Testing will be performed on both decanted oxytocin and placebo after manufacturing at Sanivo Pharma AS. The pH will be tested by means of a pH-meter. Microbiological testing will be performed according to Ph Eur 2.6.12: Microbiological examination of non-sterile products. Microbial enumeration tests. According to Ph Eur 5.1.4: Microbiological quality of non-sterile pharmaceutical preparations and substances for pharmaceutical use: Nasal use:

• Total aerobic microbial count (TAMC): Max 200 cfu/mL

• Total yeast/mold count (TYMC): Max 20 cfu/mL

• Staph aureus: absence in 1 mL

• Pseudomonas aeruginosa: absence in 1 mL

**Stability and storage**

A previous study with re-bottled Syntocinon showed no significant loss of effect of oxytocin after 6 months of storage ^18^. The stability test in this suidy was performed, to see if decanting shortened the shelf-life of Syntocinon. The type of bottles used was not specified, but the author of the article, Dr. M. Fewtrell, wrote us in an e-mail that they used white plastic bottles. We are using amber glass bottles (see specifications in appendix A), since Syntocinon is manufactured in amber glass bottles. The pump was made by various plastic materials, similar to the pump that will be used in the present study. We therefore presume that the expected shelf-life will be at least as good as in the study we referred to. We therefore set the expiration date to 6 months after re-bottleling if stored chilled (it will be ensured that all the original bottles have expiration dates of more than 6 months from the day of rebottling). One month remains as the expiration date after opening of the nasal spray container.

**Nasal spray pump and depositon**

A specification on the pump system, including the dip-tube and actuator is attached, see appendix A. The actuator has not been modified for this study. Deposition of drug in actuator is not considered relevant, since priming before use will clean the actuator for possible residues from earlier use. After longer periods without use, parts of the fluid could flow back to the bottle and be replaced by air in the pump system, but our instructions for priming immediately before each administration of the spray will handle this situation as well.

**Adsorption to plastic materials in nasal spray pump**

Regarding the possible risk of adsorption of oxytocin to plastic materials in the bottle/pump, the following considerations have been made: The bottle is made of glass, to which there is no risk of adsorption. The pump consists of the plastic materials polypropylene (PP), polyethylene (PE) and poloxymethylene (POM). We have not found any studies on the adsorption of oxytocin to these (or other) plastic materials. We have also checked for studies on the closely related polypeptide vasopressin (which also consists of 9 amino acids, only two of which differ from those of oxytocin), but there were no studies on this hormone either. However, for another peptide hormone, insulin, numerous studies have been performed on adsorption to plastic materials. These studies show that there is no risk of adsorption to materials like PP, but that adsorption to polyvinylchloride (PVC) takes place ^19,20^. Our pump does not contain PVC. Moreover, the surface area of the plastic material in the pump is considerably lower and the time period that the drug delivered to the patient is in contact with the surface of the pump system is considerably shorter that in insulin delivery systems. Taken together, these data indicate that there will not be any significant adsorption to the plastic materials affecting the oxytocin dose delivered to the patient.

To ensure that there is no significant deposistion or adsorption of oxytocin in the spray mechanism or bottle, additional testing will be performed when the production starts. The amount of oxytocin present in the first spray of decanted Syntocinon (immediately after 5 priming sprays) is measured and repeated after 1 month of storage (immedialtely after 1-3 priming sprays as needed) on two bottles of decanted Syntocinon to see if there is loss of active ingredient when the solution is in contact with the plastic parts of the nasal spray pump.

## Dosage and Drug Administration

Information on spray pattern and distribution provided by the trade agency company is attached in appendix A.

Phase 1: 3-day detoxification with 1 dose x 2 daily. Each test dose consists of 6 insufflations of Syntocinon Spray or placebo, with each insufflation given 15 sec apart and alternating between nostrils. Each insufflation consists of 6.7 μg oxytocin.The first intranasal dose of the test substances will be administrated immediately after group allocation. Patients are given instruction in proper intranasal self-administration techniques during their first dose. Instructions of priming and use is also noted in the notebook the participants are given. The second dose is taken at 1800h on admission day. The following two days (day 2-3) the patients will take their intranasal doses at 0900h and at 1800h under supervision. Patients will also be wearing an actigraph during detoxification which will register akathisia and sleep in an inpatient setting.

Phase 2: Patients completing the 3 day oxytocin or placebo detoxification trial, are discharged to a 4 week trial of self-administrated intranasal spray (as needed), with maximum use 2 insufflations x 3 daily in order to reduce alcohol cravings or consumption. Staff and patients will be instructed in priming the nasal spray by completing 5 test sprays before first use as well as 1-3 sprays later on, before administering the spray.

The following specific instructions will be given to the staff and the patients^21^:

1. Use the head upright position in standard practice but allow patients to choose the most comfortable position for themselves.

2. Do not administer if the nose is heavily congested and clear the nose from obvious obstruction before administering.

3. Ensure the bottle is primed. To do so, complete 1-3 test sprays as needed before each dose, until a fine mist can be seen and the spray is ready for use.

4. Close one nostril with one finger while administering the spray to the other nostril. Upon delivery of the medication, inhale and breathe in lightly.

5. Insert bottle 1 cm into the nostril and encourage an administration vertically into the nostril.

6. Alternate administrations between nostrils, 15 seconds between each spray.

7. Keep dust cap on whenever nasal spray is not in use.

Both the staff and the patients will be given hands-on training related to this procedure before administration of the product.

## Duration of Therapy

Use of nasal spray will last for 3 days in-patient treatment, followed by 4 weeks outpatient treatment.

Subjects may also discontinue protocol therapy in the following instances:

- Intercurrent illness which would in the judgment of the investigator effect patient safety, the ability to deliver treatment or the primary study endpoints.
- Self-reported or observed side-effects or adverse effects.
- Request by patient.

## Premedication and Monitoring

CIWA-Ar with blood pressure and heart rate monitoring are to be assessed as usual, frequency or change of monitoring necessary. See appendix B.

## Concomitant Medication

The following medication is not allowed while the patient is included in the study:

Restriction applies to chronic use.

- Any kind of benzodiazepines: Alprazolam (Xanor), Diazepam (Vival, Stesolid), Flunitrazepam (Rohypnol), Lorazepam (Temesta), Nitrazepam (Mogadon, Apodorm), Oxazepam (Sobril), Klonazepam (Rivotril), Triazolam (Halcion), benzodiazepine-like medication and z-hypnotica: Zolpidem (Stilnoct), Zopiklone (Imovane, Zopiclone).

All concomitant medication (incl. vitamins, herbal preparation and other “over-the-counter” drugs) used by the patient will be recorded in the patient’s file and CRF. At discharge, the patient will receive a notebook dated with the dates for next 4 weeks; one day per page. Here, time and dosage (including any changes of dosages) of any concomitant medication or herbal product etc. ingested will be filled in. The patient cannot participate concomitantly in another medical clinical trial. All medication will be administered to in-patients which will ensure that the study’s restrictions on use and dosages are followed, and that any adverse effect is registered in the CRF.

## Subject Compliance

Procedures for determining compliance with medications at in-patient setting will be observed by staff. Nasal spray administration 2 hours prior to or 2 hours after scheduled is not considered a deviation from compliance or time schedule. This could happen e.g. if patients are sleeping, as patients will not be awakened if they are sleeping. Nasal spray will be weighed when patients return for their last visit after 4 weeks, to estimate use. The last visit (day 30) will take place in the period day 28 -32, as close to day 30 as practically feasible. Also, the patient will receive a notebook in which the date, time and dosage of nasal spray use, use of concomitant medication and alcohol intake during the 4 weeks after discharge should be noted. The patients will also receive a follow-up phone call 1 week after discharge.

## Drug Accountability

The responsible site personnel will confirm receipt of study drug, and also keep track of number of bottles in use in a form which is kept locked inside the medicine cabinet, and will use the study drug only within the framework of this clinical study and in accordance with this protocol.

In-patients will be given drug or placebo nasal spray twice daily by nurses.The nasal spray will be kept in a medicine cabinet whenever not used and will be administered to the patients. The batch number will be noted in the CRF of the patients. Any deviation from the planned dosage is also noted in the CRF.

Outpatients will have nasal spray available at home. The number of bottles and the batch number(s) given to each patient will be noted in the CRF. When patients come back for follow-up interview, the containers will be returned to Lade Addiction Treatment Center after weighing. Lade Addiction Treatment Center will follow standard hospital procedure for destruction after use.

## Drug Labeling

The investigational product will have a label permanently affixed to the outside and will be labeled according with ICH GCP and national regulations, stating that the material is for clinical trial / investigational use only and should be kept out of reach of children.

Labels will be written in Norwegian. There is one label for in-patient use, another label for use at home. See appendix D.

## Subject Numbering

Each subject is identified in the study by a unique subject number that is assigned when subject signs the Informed Consent Form. Once assigned the subject number it cannot be reused for any other subject. The same primary identifier is used throughout the study.

The study treatment will be administered to the subject by nurses only when admitted for in-patient treatment. As outpatients the treatment will be dispensed to subjects.

# STUDY procedures

## Flow Chart

Table 1. Trial flow chart

|  | **In-patient period (day)** | | | **Outpatient Period (day)** | |
| --- | --- | --- | --- | --- | --- |
| **Time** | **On arrival**  **D 1** | **D 2** | **Discharge**  **D 3** | **D 30** | **D 60** |
| Informed consent | X |  |  |  |  |
| Inclusion/exclusion  Evaluation | X |  |  |  |  |
| Medical History, record of concomitant medication | X |  |  | X |  |
| Prior treatment | X |  |  |  |  |
| Physical Examination^1)^ | X |  |  |  |  |
| Vital signs, CIWA-Ar ^2)^ | X | X | X | X |  |
| Blood samples^3)^ | X |  | X | X |  |
| Urine samples^4)^ | X |  | X | X |  |
| Actigraph | X | X | X |  |  |
| Staff administered nasal spray | X | X | X |  |  |
| Self-reported sleep | X | X | X | X |  |
| Alcohol Craving Experience Questionnaire(ACE), SCL-10, PAM-13^5)^ |  |  | X | X |  |
| RMET^6)^, dot-probe-task^7)^ |  | X | X | X |  |
| Self-administered and reported use of nasal spray, relapse to drinking and weighing of spray bottles |  |  |  | X |  |
| Alcohol consumption (Time-Line Follow-back^8)^, Days until relapse, Daily quantity of standard alcohol units, Number of binge drinking episodes, self-reported intake of benzodiazepines |  |  |  | X |  |
| Phone interview with AUDIT^9)^ |  |  |  |  | X |

1. Cor/pulm/abdomen and peripheral lymph node status and neurology with emphasis on alcoholic damage to the cerebellum and signs of Wernicke/Korsakoffs syndrome.
2. Blood pressure, pulse, temperature, weight, alcohol expiration test until 0‰ x 2. CIWA-Ar after symptom driven protocol
3. Once daily: CRP, Hb, HCT, WBC (incl.differential counting, Na, K, Ca, glucose, creatinine, ALAT, LD, ALP, γGT, INR, albumin, bilirubin, p-oxytocin, CDT, PEth.
4. Once daily: Toxicology screening: urine dipstick and laboratory tests (incl. the ethanol metabolites EtG and EtS. (Pregnancy test (HCG) at admittance in fertile women).
5. See chapter 7.3 Other assessments
6. See chapter 7.3 Other assessments
7. See chapter 7.3 Other assessments
8. See chapter 7.3 Other assessments
9. See chapter 7.3 Other assessments

## On arrival

**Informed consent**

Informed consent must have been given voluntarily by each subject that meets the inclution and exclution criterias before any study specific procedures are initiated.

The following tests will be done at screening at admittance:

**Clinical status**

See Table 1. This physical check-up is standard procedure for all patients admitted at Lade Behandlingssenter and includes a physical examination, vital signs and history taking of alcohol intake and prior illnesses.

**Concomitant medication**

History taking of concomitant medication is standard procedure for all patients admitted at Lade Behandlingssenter. All concomitant medication (incl. vitamins, herbal preparation and other “over-the-counter” drugs) used by the subject of treatment start will be recorded in the CRF.

**Laboratory analysis**

Blood samples will be obtained at time of admittance to determine subjects’ overall clinical status (CRP, Hb, HCT, WBC (incl.differential counting, Na, K, Ca, glucose, creatinine, ALAT, LD, ALP, γGT, INR, albumin, bilirubin), p-oxytocin and toxicology (including the ethanol markers CDT and PEth – in addition to EtG and EtS in urine). Pregnancy test (HCG) is taken at admittance in fertile women. Urine dipstick is a part of standard procedure in all patients.

### Treatment Start, D1

Since patients are likely to experience alcohol abstinence, they will undergo CIWA-Ar registrations as described in section 7.2. Medication for abstinence (benzodiazepines in the form of oxazepam) is given based on CIWA-Ar-scores. Study subjects will receive the first dose of nasal spray shortly thereafter, while following the standard procedures for abstinence treatment. Study subjects receive the Actigraph to be carried throughout the stay.

### During Treatment, D1-D3

CIWA-Ar registrations are performed to monitor abstinence severity; these measturements include blood pressure and heart rate. Since CIWA-Ar is symptom driven, there are no exact times for these measurements. Nasal spray is given twice daily, at 0900h and at 1800h. The nasal spray container is weighed at the end of the inpatient period.

Day 3; at discharge: Actigraphs are handed in. Blood and urine samples are obtained. Patients are asked to fill out questionnaires at day 3, see table in section 7.3. Patients are instructed in the use of nasal spray as needed for the next 4 weeks. They will receive a notebook in which they will keep a log of use of the study drug, any intake of concomitant medication etc., and possible adverse events.

### End of Treatment and End of Study Visit, D 30

Clinical status with vital signs and laboratory tests is repeated. Patients fill out questionnaires (see table in section 7.3), and hand in nasal sprays and their notebooks. Nasal spray containers are weighed. A phone interview will be done at day 60.

### Withdrawal Visit

If the patient is withdrawn from the study before completing the study by his/her own will, 3 attempts to reach subject for is made (e.g telephone, mail).Patients withdrawing from the study will go back to standard procedures for the follow-up treatment at Lade Behandlingssenter. Because of no expected side effects and the short half-life of oxytocin, no further examinations or tests are considered needed. The nasal sprays are to be returned to Lade Behandlingssenter.

## Criteria for Patient Discontinuation

Patients may be discontinued from study treatment and assessments at any time. Specific reasons for discontinuing a patient for this study are:

- Voluntary discontinuation by the patient who is at any time free to discontinue his/her participation in the study, without prejudice to further treatment.
- Safety reason as judged by the Principal Investigator
- Major protocol deviation, which would be not following guidelines of CIWA-Ar and medication or not following guidelines for nasal spray doses.
- Incorrect enrolment ie, the patient does not meet the required inclusion/exclusion criteria for the study
- Patient lost to follow-up
- A female patient becoming pregnant
- Deterioration in the patients condition which in the opinion of the Principal Investigator warrants study medication discontinuation (to be records as an AE or under Investigator Discretion)
- Patient’s non-compliance to study treatment and/or procedures

## Procedures for Discontinuation

### Patient Discontinuation

Patients who withdraw or are withdrawn from the study, will discontinue treatment with oxytocin. They will however continue their treatment for alcohol addiction at Lade Addiction Treatment Center. If possible, a final assessment will be performed (end of study visit or by phone). The reason for discontinuation will be recorded, if the patient gives any. The investigator is obliged to follow up any significant adverse events until the outcome either is recovered or resolved, recovering/resolving, not recovered/not resolved, recovered/resolved with sequelae, fatal or unknown.

Patient`s who withdraw or are withdrawn from the study before start of treatment, will be replaced.

### Trial Discontinuation

The whole trial may be discontinued at the discretion of the PI or the sponsor in the event of any of the following:

- Occurrence of AEs unknown to date in respect of their nature, severity and duration
- Medical or ethical reasons affecting the continued performance of the trial
- Difficulties in the recruitment of patients

The sponsor and principal investigator will inform all investigators, the relevant Competent Authorities and Ethics Committees of the termination of the trial along with the reasons for such action. If the study is terminated early on grounds of safety, the Competent Authorities and Ethics Committees will be informed within 15 days.

## Laboratory Tests

Bloodsamples, urine samples: Sample collection, handling and analysis will be performed in accordance with hospital/laboratory standard procedures at St. Olavs Hospital.

# assessments

## Assessment of Efficacy Response

Efficacy response is measured as the total number of mg of oxazepam used by study subjects undergoing detoxification based on a symptom-driven protocol using CIWA-Ar scores (see Section 7.2). The dosages of oxazepam and the CIWA-Ar-scores are registered in the CRF.

Actigraph-assessed akathisia and self-reported sleep will also be compared with CIWA-Ar scores and oxazepam dosages retrospectively.

## Safety and Tolerability Assessments

Safety will be monitored by the assessments described below as well as the collection of AEs at every visit. Significant findings that are present prior to the signing of informed consent must be included in the relevant medical history/ current medical condition page of the CRF. For details on AE collection and reporting, refer to Section 8.4.

For the assessment schedule refer to Flow chart in Section 6.1.

**CIWA-Ar**

CIWA-Ar has 10-items assessing headache, visual and tactile and auditory disturbances, paroxysmal sweats, orientation and clouding, agitation and anxiety (all assessed on a 0-7 severity scale). It also includes blood pressure, pulse, respiration rate, temperature. A symptom-driven protocol of PRN administrations of oxazepam will be followed to all included patients while undergoing detoxification. Symptom-triggered benzodiazepine treatment has been shown to require less total medication compared to fixed regimen benzodiazepine tapers ^22^. A ‘‘standing order set’’ to administer oxazepam based on a patient’s CIWA-Ar score will as such be followed. Mild CIWA-Ar scores (0 to 9)^3^ indicate mild withdrawal symptoms and indicate no benzodiazepine treatment (reassessed in 4 hours). A CIWA-Ar score of 10 to 14 represents moderate withdrawal and 10 mg oxazepam will be given orally as needed, scores reassessed after 1 hour. Severe withdrawal is represented by a score 15 and above and indicate oxazepam 15 mg given orally as needed, scores reassessed after 1 hour. All benzodiazepines (dosage and amount) will be documented along with CIWA-scores.

Laboratory evaluation:

St. Olav’s University Hospital, department of medical biochemistry will be used for the analysis of hematology and biochemistry specimens collected, except plasma-oxytocin which is sent to the Hormonlaboratoriet at Oslo Universitetssykehus.

Hematology assessments will include the following parameters: hemoglobin, platelets, CRP, HCT, WBC (incl.differential counting,

Blood chemistry assessments will include the following: albumin, creatinine, Na, K, Ca, glucose, ALAT, LD, ALP, γGT, INR, bilirubin, p-oxytocin, CDT, PEth, HCG (in fertile women).

Urine analysis will include a toxicological screening including ethanol metabolites - EtG and EtS.

## Other Assessments

Summary of measures by assessment point

**SCL-10 (Symptom check list -10)**

A tool used to uncover psychiatric symptoms of depression and anxiety. The questionnaire is translated to Norwegian and validated in a Norwegian population^23^.

**Time-Line Follow-back**

A visual tool helping patients to remember events the previous 30 days ^24^.

**Alcohol Craving Experience Questionnaire (ACE)**

One of the most used tools to measure craving of alcohol ^25^. There is no Norwegian translation available; therefore this questionnaire has been translated into Norwegian by our research group for this purpose before study start.

**PAM-13 (Patient activation measure -13)**

13 questions measuring the patient’s ability to acknowledge and take control of own health issues. The questionnaire is translated to Norwegian and validated in population with both chronic disease and pshyiatric disorders ^26,27^

**Tests performed on computer approximately 40 minutes after administration of nasal spray:**

**RMET (Reading the mind in the eye test)**

Contains 36 images of the eye region of different faces. Patients will try to find the corresponding word by choosing between four response options for each image. We used a split version. Day 2: 18 pictures, day 3: 18 pictures, day 30: 36 pictures. ^28,29^

**Dot-probe test**

A test used to measuring attentional bias. Patients look at different face pairs comprising an emotional face (angry or happy) paired with a neutral face for a duration of 500 ms, before a probe replaces the location. Participants will try to press a key on the keyboard that correspond to the location of the probe as soon as possible after it appears. The tes tis repeated on day 2, day 3 and day 30. ^30,31^

**AUDIT (alohol use disorder identification test)**

A 10-item screening tool developed by the World Health Organization (WHO) to

assess alcohol consumption, drinking behaviors, and alcohol-related

problems. Patients will be nterviewed on the phone at Day 60 regarding the last 30 days.^32^

# Safety MONItoring and reporting

The investigator is responsible for the detection and documentation of events meeting the criteria and definition of an adverse event (AE) or serious adverse event (SAE). Each patient will be instructed to contact the investigator immediately should they manifest any signs or symptoms they perceive as serious.

The methods for collection of safety data are described below.

## Definitions

### Adverse Event (AE)

An AE is any untoward medical occurrence in a patient administered a pharmaceutical product and which does not necessarily have a causal relationship with this treatment.

An adverse event (AE) can therefore be any unfavorable and unintended sign (including an abnormal laboratory finding), symptom, or disease temporally associated with the use of a medicinal (investigational) product, whether or not related to the medicinal (investigational) product.

The term AE is used to include both serious and non-serious AEs.

. If an abnormal laboratory value/vital sign are associated with clinical signs and symptoms, the sign/symptom should be reported as an AE and the associated laboratory result/vital sign should be considered additional information that must be collected on the relevant CRF.

### Serious Adverse Event (SAE)

Any untoward medical occurrence that at any dose:

- Results in death
- Is immediately life-threatening
- Requires in-patient hospitalization or prolongation of existing hospitalization
- Results in persistent or significant disability or incapacity
- Is a congenital anomaly/birth defect
- Is an important medical event that may jeopardize the subject or may require medical intervention to prevent one of the outcomes listed above.

Medical and scientific judgment is to be exercised in deciding on the seriousness of a case. Important medical events may not be immediately life-threatening or result in death or hospitalization, but may jeopardize the subject or may require intervention to prevent one of the listed outcomes in the definitions above. In such situations, or in doubtful cases, the case should be considered as serious. Hospitalization for administrative reason (for observation or social reasons) is allowed at the investigator’s discretion and will not qualify as serious unless there is an associated adverse event warranting hospitalization.

### Suspected Unexpected Serious Adverse Reaction (SUSAR)

Adverse Reaction: all untoward and unintended responses to an investigational medicinal product related to any dose administered;

Unexpected Adverse Reaction: an adverse reaction, the nature or severity of which is not consistent with the applicable product information.

Suspected Unexpected Serious Adverse Reaction: SAE (see section 8.1.2) that is unexpected as defined in section 8.2 and possibly related to the investigational medicinal product(s).

## Expected Adverse Events

Side-effects described by NOMA:

(≥1/10 000, <1/1000): headache, nausea, allergic dermatitis (≥1/1000, <1/100): uterus contractions.

Side-effects by use of Oxytocin are also usual sympthoms patients undergoing detoxification on alcohol are likely to experience. Trained medical staff will observe changes in patient’s wellbeing when monitoring their withdrawal sympthoms, and are available at all times for questions.

Syntocinon nasal spray in the amount used is not known to give any discomfort or known side effects. Half-life of oxytocin is also short; 2-20 minutes. All exess nasal spray will be eliminated in the gastrointestinal channel.

Epileptic seizures, delirium tremens may occur in severe alcohol withdrawal, and will not be considered as a SAE.

## Time Period for Reporting AE and SAE

For each patient the standard time period for collecting and recording AE and SAEs will begin at start of study treatment and will continue following the last dose of study treatment is given, because of the short half-life of oxytocin.

During the course of the study all AEs and SAEs will be proactively followed up for each patient; events should be followed up to resolution, unless the event is considered by the investigator to be unlikely to resolve due to the underlying disease. All patients will receive oxytocin or placebo during three days as in-patients supervised by medical staff at all times.

## Recording of Adverse Events

If the patient has experienced adverse event(s), the investigator will record the following information in the CRF:

- The nature of the event(s) will be described by the investigator in precise standard medical terminology (i.e. not necessarily the exact words used by the patient).
- The duration of the event will be described in terms of event onset date and event ended data.
- The intensity of the adverse event: Mild / Moderate / Severe; according to Common Terminology Criteria for Adverse Events version 4.0 (CTCAE).
- The Causal relationship of the event to the study medication will be assessed as one of the following:

Unrelated:

There is not a temporal relationship to investigational product administration (too early, or late, or investigational product not taken), or there is a reasonable causal relationship between non-investigational product, concurrent disease, or circumstance and the AE.

Unlikely:

There is a temporal relationship to investigational product administration, but there is not a reasonable causal relationship between the investigational product and the AE.

Possible:

There is reasonable causal relationship between the investigational product and the AE. Dechallenge information is lacking or unclear.

Probable:

There is a reasonable causal relationship between the investigational product and the AE. The event responds to dechallenge. Rechallenge is not required.

Definite:

There is a reasonable causal relationship between the investigational product and the AE.

- Action taken
- The outcome of the adverse event – whether the event is resolved or still ongoing.

It is important to distinguish between serious and severe AEs. Severity is a measure of intensity whereas seriousness is defined by the criteria in Section 8.1. An AE of severe intensity need not necessarily be considered serious. For example, nausea that persists for several hours may be considered severe nausea, but is not an SAE. On the other hand, a stroke that results in only a limited degree of disability may be considered a mild stroke, but would be an SAE.

## Reporting Procedure

### AEs and SAEs

All adverse events and serious adverse events that should be reported as defined in section 8.1.1 will be recorded in the patient's CRF.

SAEs must be reported by the investigator to the sponsor, within 24 hours after the site has gained knowledge of the SAE. Every SAE must be documented by the investigator on the SAE pages (to be found as part of the CRF). The Serious Adverse Event Report Form must be completed, signed and sent to the sponsor. The initial report shall promptly be followed by detailed, written reports if necessary. The initial and follow-up reports shall identify the trial subjects by unique code numbers assigned to the latter.

The sponsor keeps detailed records of all AEs reported by the investigators and performs an evaluation with respect to seriousness, causality and expectedness. Investigators will if necessary exclude patient from the trial.

### SUSARs

SUSARs will be reported to the Competent Authority and Ethics Committee according to national regulation. The following timelines should be followed.

The sponsor will ensure that all relevant information about suspected serious unewpeted adverse reactions that are fatal or life-threatening is recorded and reported as soon as possible to the Competent Authority and Ethics ommittee in any case no later than seven (7) days after knowledge by the sponsor of such a case, and hat relevant follow-up information is subsequently communicated within an additional eight (8) days.

All other suspected serious unexpected adverse reactions will be reported to the Comptent Authority concerned and to the Ethics Comitee concerned as soon as possible, but within a maximum of fifteen (15) days of first knowledge by the sponsor.

SUSAR will be reported using the CIOMS form from www.legemiddelverket.no.

### Annual Safety Report

Once a year throughout the clinical trial, the sponsor will provide the Competent Authority with an annual safety report. The format will comply with national requirements.

### Clinical Study Report

The adverse events and serious adverse events occurring during the study will be discussed in the safety evaluation part of the Clinical Study Report.

## Procedures in Case of Emergency

The investigator is responsible for assuring that there are procedures and expertise available to cope with emergencies during the study.

If a patient experiences a SUSAR (see above), unblinding will be perfomed immediately, and before it is reported to the Competent Authority. To avoid unblinding of other study patients, information regarding whether the patient has received oxytocin or placebo will be kept in separate sealed envelopes, one for each patient with the drug-package numberwritten on the outside. The envelopes will be kept in a locked cabinet in the treatment facility. If necessary, the nurse on duty will contact the primary investigator or his representant at Lade Behandlingssenter to get knowledge of the location of the envelopes, open the envelope with the patient’s study ID, and unblind the patient.

# Data management and monitoring

## Case Report Forms (CRFs)

The designated investigator or staff will enter the data required by the protocol into the Case report forms (CRF). The Principal Investigator is responsible for assuring that data entered into the CRF is complete, accurate, and that entry is performed in a timely manner. If any assessments are omitted, the reason for such omissions will be noted on the CRFs. Corrections, with the reason for the corrections will also be recorded.

After database lock, the investigator will receive paper copies of the subject data for archiving at the investigational site.

Type of CRF used: WebCRF.

## Source Data

The medical records for each patient should contain information which is important for the patient’s safety and continued care, and to fulfill the requirement that critical study data should be verifiable.

To achieve this, the medical records of each patient should clearly describe at least:

- That the patient is participating in the study, e.g. by including the enrollment number and the study code or other study identification;
- Date when Informed Consent was obtained from the patient and statement that patient received a copy of the signed and dated Informed Consent;
- Results of all assessments confirming a patient’s eligibility for the study;
- Diseases (past and current; both the disease studied and others, as relevant);
- Surgical history, as relevant;
- Treatments withdrawn/withheld due to participation in the study;
- Results of assessments performed during the study, including CIWA-scores;
- Treatments given, changes in treatments during the study and the time points for the changes;
- Number of nasal spray bottles;
- Visits to the clinic / telephone contacts during the study, including those for study purposes only;
- Non-Serious Adverse Events and Serious Adverse Events (if any) including causality assessments;
- Date of, and reason for, discontinuation from study treatment;
- Date of (and reason for) withdrawal from study;
- Date of death and cause of death, if available;
- Additional information according to local regulations and practice.

The records for each subject should contain information which is important for the subject’s safety and to fulfill the requirement that critical study data should be verifiable.

## Study Monitoring

A clinical study monitor will visit the investigator in a regular basis according to the monitor plan. A monitoring plan specific to this study is held separately to the protocol. The monitoring plan outlines the level of monitoring to be performed for the trial and how this will be carried out. The level of monitoring is based on the phase of the trial and any perceived risks. Monitoring visits will commence after the Sponsor approves the plan.

Monitor at AKF, NTNU and the study team has agreed that the study is going to be monitored according to a medium risk. This means that 20 % of all data entered in the study CRF is going to be source data verified, all patient informed consents will be controlled, All SAEs and the primary endpoint in the study will also be fully monitored.

ISF/TMF will be monitored and the monitor will confirm that the study is conducted in compliance with ICH-GCP, approved research protocol and other relevant laws and regulations. Study drug accountability will also be monitored.

## Confidentiality

The investigator shall arrange for the secure retention of the patient identification and the code list. Patient files shall be kept for the maximum period of time permitted by each hospital. The study documentation (CRFs, Site File etc) shall be retained and stored during the study and for 15 years after study closure. All information concerning the study will be stored in a safe place inaccessible to unauthorized personnel.

## Database management

When enrolled in the project, patients will have a study form with both name/national identity code and study ID. At discharge, these forms connecting the patient’s identity to the Study ID will be kept separately, and the data with only Study ID as identifying code will be transformed to a secondary study form containing only the Study ID for further analysis. The data linking patient ID to study ID will be transferred to the Unit of Applied Clinical Research for storage in accordance with national regulations, for 15 years after the termination of the study. Randomisation code is kept with the Unit of Applied Clinical Research until code can be broken.

# Statistical methods and data analysis

## Determination of Sample Size

In a previous study in patients receiving intranasal oxytocin ^17^, the mean dose lorazepam given was 3.4 mg in the oxytocin group and 16.5 mg in the control group. These doses correspond to about 60-70 mg oxazepam and about 320-340 mg oxazepam, respectively. The difference found in that study as well as the does used are, however, rather extreme, and far outside the doses used in both in Norway in general and at Lade Addiction Treatment Center. At Lade Addiction Treatment Center, the oxazepam doses generally range from 0 to 60 mg, with an estimated mean of about 20-30 mg and a SD of about 10 mg. As a difference of 10 mg in oxazepam consumption is of clinical interest to detect between groups, 16 patients in each group are required given an alpha value of 0.05 and a power of 80 %. Therefore, a total of 40 patients will be included in the study.

## Randomization

Randomization will be performed by Unit of Applied Clinical Research, NTNU.

### Allocation- procedure to randomize a patient

Preparation of active drug or placebo will produce identical nasal sprays. Who to receive the active drug will be determined by electronic randomization executed with WebCRF, provided by the Unit of Applied Clinical Research at the Norwegian University of Science and Technology (NTNU). More information on the randomization services from the Unit of Applied Clinical Research can be found at <http://www.ntnu.edu/dmf/akf/randomisering>.

### Blinding and emergency unblinding

Patient, investigator, personnel assessing outcomes and data analyst are blinded during this study. The randomization will be done electronically by the nurse on duty which will have an instruction sheet for further administration of the spray compound. The randomization prosess provides a drug package number corresponding with the Study ID-number of the patient. Neither the clinicians/staff nor the patients will know which spray bottles contain the active drug. The protocol linking the ID-numbers to the randomization will be kept at the Unit of Applied Clinical Research and will not be revealed to the researchers until a study arm is completed. In case of emergency, unblinding will be perfomed immediately and before it is reported to the Authority. To avoid unblinding of other study patients as well, information regarding whether each patient has received oxytocin or placebo will be kept in separate sealed envelopes, one for each patient. See also section 8.6.

## Population for Analysis

The following populations will be considered for the analyses:

- Intention to treat (ITT) population: Includes all randomized participants, regardless of protocol adherence.
- Per-protocol population (PP): Includes all randomized participants showing full protocol adherence.
- Safety population: Includes all subjects who have received at least one dose of study medication. Subjects who withdraw from the study will be included in the safety analysis. A list of withdrawn subjects, preferably with the reasons for withdrawal, will be made.

## Statistical Analysis

Statistical analysis will be performed by Unit for Applied Research, NTNU.

Descriptive data will be presented as mean ± SD. Dichotomuos outcome variables will be compared using non-parametric test (paired Wilcoxon signed rank test, and McNemar´s test). P-values <0.05 will be considered significant.

0-hypothesis: Oxytocin nasal spray does not differ from placebo in reducing alcohol withdrawal symptoms or decrease craving after detoxification

Primary hypothesis 1: Oxytocin nasal spray can reduce the amount of oxazepam needed to complete 3 days of alcohol detoxification.

Primary hypothesis 2: Oxytocin nasal spray can decrease craving the subsequent 4 weeks after detoxification.

Group comparisons of oxazepam use (measures in mg) at 3 days will be done with student t-test. We will use mixed‐effects regression analyses to examine the change in sleep and actigraph scores from D1 to D3, and changes in anxiety, depression and alcohol use from discharge (D3) to follow-up (D30). Statistics will be performed with per-protocol and intention-to-treat data.Mixed methods take the correlation among repeated measures into account, and they use all available data, thereby lessening concerns about missing data. The basic model will include group (oxytocin, placebo) as the between‐subjects factor and time as the repeated measure. The treatment effect will be evaluated as the group by time interaction, according to the planned comparisons. Both group and time will be fixed‐effects. Covariates can also be added to the models, in order to partial out their effects before evaluating the treatment effects. Follow‐up t‐tests and other post hoc methods will be used to test more time‐ and group‐specific hypotheses. We will test primary hypotheses at the .05 level of significance, but follow‐up and exploratory analyses will be tested at the .01 level of significance.

# STUDY MANAGEMENT

## Investigator Delegation Procedure

The principal investigator is responsible in making and updating a “delegation of tasks” listing all the involved co-workers and their role in the project. He will ensure that appropriate training relevant to the study is given to all of these staff, and that any new information of relevance to the performance of this study is forwarded to the staff involved.

## Protocol Adherence

Investigators ascertain they will apply due diligence to avoid protocol deviations.

All significant protocol deviations will be recorded and reported in the Clinical Study Report (CSR), and in the patient’s CRF or Investigator Site File (ISF) as suitable, following guidelines listed in: http://www.norcrin.no/documents/2013/05/note-to-file.pdf

## Study Amendments

If it is necessary for the study protocol to be amended, the amendment and/or a new version of the study protocol (Amended Protocol) must be notified to and approved by the Competent Authority and the Ethics Committee according to EU and national regulations.

## Audit and Inspections

Authorized representatives of a Competent Authority and Ethics Committee may visit the center to perform inspections, including source data verification. Likewise the representatives from sponsor may visit the center to perform an audit. The purpose of an audit or inspection is to systematically and independently examine all study-related activities and documents to determine whether these activities were conducted, and data were recorded, analyzed, and accurately reported according to the protocol, Good Clinical Practice (ICH GCP), and any applicable regulatory requirements. The principal investigator will ensure that the inspectors and auditors will be provided with access to source data/documents.

# Ethical and regulatory requirements

The study will be conducted in accordance with ethical principles that have their origin in the Declaration of Helsinki and are consistent with Good Clinical Practice (ICH GCP) and applicable regulatory requirements. Registration and storage of patient data will be carried out in accordance with Norwegian Personal Data Act.

## Ethics Committee Approval

The study protocol, including the patient information and informed consent form to be used, must be approved by the regional ethics committee before enrolment of any patients into the study.

The investigator is responsible for informing the ethics committee of any serious and unexpected adverse events and/or major amendments to the protocol as per national requirements.

## Other Regulatory Approvals

The protocol will be submitted and approved by the NOMA before commencement of the study.

The protocol will also be registered in www.clinicaltrials.gov before inclusion of the first patient.

## Informed Consent Procedure

The investigator is responsible for giving the patients full and adequate verbal and written information about the nature, purpose, possible risk and benefit of the study. They will be informed as to the strict confidentiality of their patient data, but that their medical records may be reviewed for trial purposes by authorized individuals other than their treating physician.

It will be emphasized that the participation is voluntary and that the patient is allowed to refuse further participation in the protocol whenever she/he wants. This will not prejudice the patient’s subsequent care. Documented informed consent must be obtained for all patients included in the study before they are registered in the study. This will be done in accordance with the national and local regulatory requirements. The investigator is responsible for obtaining signed informed consent.

A copy of the patient information and consent will be given to the patients. The signed and dated patient consent forms will be filed in the Investigator Site File binder.

## Subject Identification

The investigator is responsible for keeping a list of all patients (who have received study treatment or undergone any study specific procedure) including patient’s date of birth and national identity code, full names and last known addresses.

The patients will be identified in the CRFs by patient number.

# Trial sponsorship and financing

Funds to cover running costs of 250 000 NOK was granted by St. Olav University Hospital March 2016. Funds to cover 200 000 NOK for nurse in a 50% position was granted by the Faculty of Medicine, NTNU June 2016. Excess costs will be coverend by grants available at Department of Clinical Pharmacology, St. Olav University Hospital.

# Trial insurance

The study has insurance coverage through membership of the Drug Liability Association.

# Publication policy

Upon study completion and finalization of the study report, the results of this study will either be submitted for publication and/or posted in a publicly assessable database of clinical study results.

The results of this study will also be submitted to the Competent Authority and the Ethics Committee according to EU and national regulations.

All personnel who have contributed significantly with the planning and performance of the study (Vancouver convention 1988) may be included in the list of authors.

# REFERENCES

1 Stevens, G., Mascarenhas, M. & Mathers, C. Global health risks: progress and challenges. *Bull World Health Organ* **87**, 646 (2009).

2 Amato, L., Minozzi, S. & Davoli, M. Efficacy and safety of pharmacological interventions for the treatment of the Alcohol Withdrawal Syndrome. *Cochrane Database Syst Rev*, CD008537, doi:10.1002/14651858.CD008537.pub2 (2011).

3 Sullivan, J. T., Sykora, K., Schneiderman, J., Naranjo, C. A. & Sellers, E. M. Assessment of alcohol withdrawal: the revised clinical institute withdrawal assessment for alcohol scale (CIWA-Ar). *Br J Addict* **84**, 1353-1357 (1989).

4 McGregor, I. S. & Bowen, M. T. Breaking the loop: oxytocin as a potential treatment for drug addiction. *Hormones and behavior* **61**, 331-339, doi:10.1016/j.yhbeh.2011.12.001 (2012).

5 Buisman-Pijlman, F. T. *et al.* Individual differences underlying susceptibility to addiction: Role for the endogenous oxytocin system. *Pharmacol Biochem Behav* **119**, 22-38, doi:10.1016/j.pbb.2013.09.005 (2014).

6 Rehme, M., Hillemacher, T. & Heberlein, A. Comment on "intranasal oxytocin blocks alcohol withdrawal in human subjects" by Pedersen and colleagues (2013). *Alcoholism, clinical and experimental research* **37**, 720-721, doi:10.1111/acer.12147 (2013).

7 Forsling, M. L. *et al.* The effect of 3,4-methylenedioxymethamphetamine (MDMA, 'ecstasy') and its metabolites on neurohypophysial hormone release from the isolated rat hypothalamus. *Br J Pharmacol* **135**, 649-656, doi:10.1038/sj.bjp.0704502 (2002).

8 Bedi, G., Phan, K. L., Angstadt, M. & de Wit, H. Effects of MDMA on sociability and neural response to social threat and social reward. *Psychopharmacology (Berl)* **207**, 73-83, doi:10.1007/s00213-009-1635-z (2009).

9 Born, J. *et al.* Sniffing neuropeptides: a transnasal approach to the human brain. *Nat Neurosci* **5**, 514-516, doi:10.1038/nn849 (2002).

10 Domes, G. *et al.* Oxytocin attenuates amygdala responses to emotional faces regardless of valence. *Biol Psychiatry* **62**, 1187-1190, doi:10.1016/j.biopsych.2007.03.025 (2007).

11 Kirsch, P. *et al.* Oxytocin modulates neural circuitry for social cognition and fear in humans. *J Neurosci* **25**, 11489-11493, doi:10.1523/JNEUROSCI.3984-05.2005 (2005).

12 de Oliveira, D. C., Zuardi, A. W., Graeff, F. G., Queiroz, R. H. & Crippa, J. A. Anxiolytic-like effect of oxytocin in the simulated public speaking test. *J Psychopharmacol* **26**, 497-504, doi:10.1177/0269881111400642 (2012).

13 Pedersen, C. A. *et al.* Intranasal oxytocin reduces psychotic symptoms and improves Theory of Mind and social perception in schizophrenia. *Schizophr Res* **132**, 50-53, doi:10.1016/j.schres.2011.07.027 (2011).

14 MacDonald, E. *et al.* A review of safety, side-effects and subjective reactions to intranasal oxytocin in human research. *Psychoneuroendocrinology* **36**, 1114-1126, doi:10.1016/j.psyneuen.2011.02.015 (2011).

15 Lee, H. J., Macbeth, A. H., Pagani, J. H. & Young, W. S., 3rd. Oxytocin: the great facilitator of life. *Prog Neurobiol* **88**, 127-151, doi:10.1016/j.pneurobio.2009.04.001 (2009).

16 Jodogne, C., Tirelli, E., Klingbiel, P. & Legros, J. J. Oxytocin attenuates tolerance not only to the hypothermic but also to the myorelaxant and akinesic effects of ethanol in mice. *Pharmacol Biochem Behav* **40**, 261-265 (1991).

17 Pedersen, C. A. *et al.* Intranasal oxytocin blocks alcohol withdrawal in human subjects. *Alcoholism, clinical and experimental research* **37**, 484-489, doi:10.1111/j.1530-0277.2012.01958.x (2013).

18 Fewtrell, M. S., Loh, K. L., Blake, A., Ridout, D. A. & Hawdon, J. Randomised, double blind trial of oxytocin nasal spray in mothers expressing breast milk for preterm infants. *Arch Dis Child Fetal Neonatal Ed* **91**, F169-174, doi:10.1136/adc.2005.081265 (2006).

19 Seifi, A., Mowla, A., Vaziri, M. M., Talei, A. R. & Namazy, M. R. Insulin adsorbance to polyvinylchloride (PVC) surfaces of fluid container and infusion-set. *Middle East J Anaesthesiol* **17**, 975-981 (2004).

20 Thompson, C. D., Vital-Carona, J. & Faustino, E. V. The effect of tubing dwell time on insulin adsorption during intravenous insulin infusions. *Diabetes Technol Ther* **14**, 912-916, doi:10.1089/dia.2012.0098 (2012).

21 Guastella, A. J. *et al.* Recommendations for the standardisation of oxytocin nasal administration and guidelines for its reporting in human research. *Psychoneuroendocrinology* **38**, 612-625, doi:10.1016/j.psyneuen.2012.11.019 (2013).

22 Daeppen, J. B. *et al.* Symptom-triggered vs fixed-schedule doses of benzodiazepine for alcohol withdrawal: a randomized treatment trial. *Arch Intern Med* **162**, 1117-1121 (2002).

23 Strand, B. H., Dalgard, O. S., Tambs, K. & Rognerud, M. Measuring the mental health status of the Norwegian population: a comparison of the instruments SCL-25, SCL-10, SCL-5 and MHI-5 (SF-36). *Nord J Psychiatry* **57**, 113-118, doi:10.1080/08039480310000932 (2003).

24 Sobell, L. C., Sobell, M. B., Leo, G. I. & Cancilla, A. Reliability of a timeline method: assessing normal drinkers' reports of recent drinking and a comparative evaluation across several populations. *Br J Addict* **83**, 393-402 (1988).

25 Statham, D. J. *et al.* Measuring alcohol craving: development of the Alcohol Craving Experience questionnaire. *Addiction* **106**, 1230-1238, doi:10.1111/j.1360-0443.2011.03442.x (2011).

26 Lara-Cabrera, M. L. *et al.* The effect of a brief educational programme added to mental health treatment to improve patient activation: A randomized controlled trial in community mental health centres. *Patient Educ Couns*, doi:10.1016/j.pec.2015.11.028 (2015).

27 Moljord, I. E. *et al.* Psychometric properties of the Patient Activation Measure-13 among out-patients waiting for mental health treatment: A validation study in Norway. *Patient Educ Couns* **98**, 1410-1417, doi:10.1016/j.pec.2015.06.009 (2015).

28 Baron-Cohen, S., Wheelwright, S., Hill, J., Raste, Y. & Plumb, I. The "Reading the Mind in the Eyes" Test revised version: a study with normal adults, and adults with Asperger syndrome or high-functioning autism. *J Child Psychol Psychiatry* **42**, 241-251 (2001).

29 Iorfino, F., Alvares, G. A., Guastella, A. J. & Quintana, D. S. Cold Face Test-Induced Increases in Heart Rate Variability Are Abolished by Engagement in a Social Cognition Task. *Journal of Psychophysiology* **30**, 38-46, doi:10.1027/0269-8803/a000152 (2016).

30 MacLeod, C., Mathews, A. & Tata, P. Attentional bias in emotional disorders. *J Abnorm Psychol* **95**, 15-20 (1986).

31 Price, R. B. *et al.* Empirical recommendations for improving the stability of the dot-probe task in clinical research. *Psychol Assess* **27**, 365-376, doi:10.1037/pas0000036 (2015).

32 Saunders, J. B., Aasland, O. G., Babor, T. F., De La Fuente, J. R. & Grant, M. Development of the Alcohol Use Disorders Identification Test (AUDIT): WHO Collaborative Project on Early Detection of Persons with Harmful Alcohol Consumption-II. *Addiction* **88**, 791-804, doi:10.1111/j.1360-0443.1993.tb02093.x (1993).

# LIST OF APPENDICES

A Specifications of nasal spray pump and sprayquality

B CIWA-Ar Guidelines

C Placebo decleration list

D Example of labeling (1. For use at home 2. For use at Lade Behandlingssenter)

E Trial insurance

# Appendix A – Specifiations of nasal spray pump and spray quality


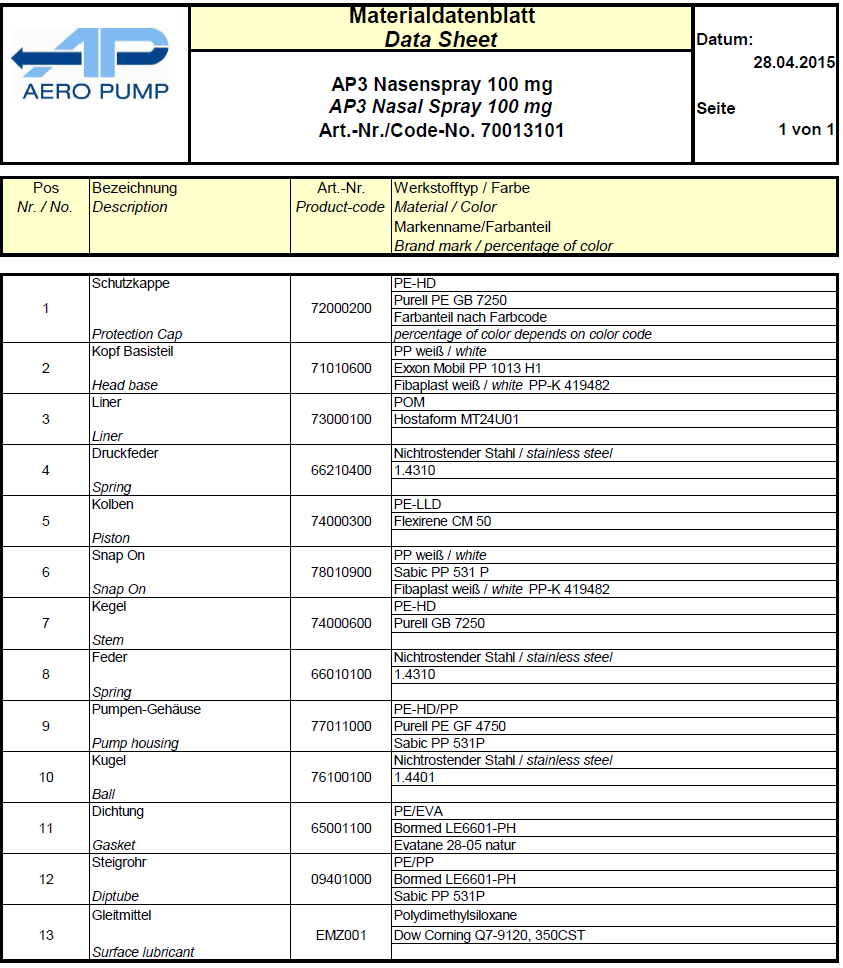


**

# Appendix B – CIWA-Ar guidelines

**CIWA-Ar**

**Clinical Institute Withdrawal Assessment -Alcohol revised**

Navnelapp

Puls eller hjertefrekvens, tatt i ett minutt.

Blodtrykk.

______________________________________________

**KVALME OG OPPKAST**

*Spør: "Føler du deg kvalm?Har du kastet opp?" Observasjon*.

0 Ikke kvalme og ikke oppkast

1 Lett kvalme uten oppkast

2

3

4 Periodisk kvalme med brekninger

5

6

7 Vedvarende kvalme, hyppige brekninger og oppkast

______________________________________________

**TREMOR**

*Armene utstrakt med spredte fingre. Observasjon*.

0 Ingen tremor

1 Ikke synlig, men kan kjennes fingertupp mot

fingertupp

2

3

4 Moderat, med pasientens armer utstrakt

5

6

7 Sterk, selv når armene ikke er utstrakt

______________________________________________

**SVETTETOKTER**

*Observasjon*.

0 Ingen synlig svette

1 Knapt merkbar svetting, klamme håndflater

2

3

4 Tydelige svetteperler i pannen

5

6

7 Gjennomvåt av svette

______________________________________________

**ANGST**

*Spør: "Føler du deg nervøs?" Observasjon*.

0 Ingen angst, rolig

1 Lett engstelig

2

3

4 Moderat engstelig eller vaktsom (slik at det oppfattes

som angst)

5

6

7 Tilsvarende akutt panikktilstand som ved alvorlig

delirium eller akutte schizofrene reaksjoner

______________________________________________

**URO**

Observasjon:

0 Normal aktivitet

1 Noe mer enn normal aktivitet

2

3

4 Moderat urolig og rastløs

5

6

7 Vandrer frem og tilbake under mesteparten av

intervjuet eller kaster på seg hele tiden

______________________________________________

______________________________________________

**SENSIBILITETSFORSTYRRELSER**

*Spør: "Har du noe form for kløe, prikking, stikking,*

*brennende/sviende følelse, nummenhet, eller kjenner du at*

*småkryp kravler på eller under huden din?" Observasjon*.

0 Ingen

1 Svært lett kløe, prikking, stikking, svie eller

nummenhet

2 Lett kløe, prikking, stikking, svie eller nummenhet

3 Moderat kløe, prikking, stikking, svie eller

nummenhet

4 Moderate hallusinasjoner

5 Sterke hallusinasjoner

6 Svært sterke hallusinasjoner

7 Vedvarende hallusinasjoner

______________________________________________

**HØRSELSFORSTYRRELSER**

*Spør: "Er du mer oppmerksom på lyder rundt deg? Er de*

*ubehagelige? Skremmer de deg? Hører du noe som er*

*urovekkende? Hører du ting som du vet ikke er der?"*

*Observasjon.*

0 Ingen

1 Svært lett ubehag eller evne til å skremme

2 Lett ubehag eller evne til å skremme

3 Moderat ubehag eller evne til å skremme

4 Moderate hallusinasjoner

5 Sterke hallusinasjoner

6 Svært sterke hallusinasjoner

7 Vedvarende hallusinasjoner

______________________________________________

**SYNSFORSTYRRELSER**

*Spør: "Virker lyset sterkere enn vanlig? Oppleves farger*

*annerledes enn vanlig? Gjør det vondt for øynene? Ser du noe*

*som er urovekkende? Ser du ting som du vet ikke er der?"*

*Observasjon*.

0 Ingen

1 Svært lett lysfølsomhet

2 Lett lysfølsomhet

3 Moderat lysfølsomhet

4 Moderate hallusinasjoner

5 Sterke hallusinasjoner

6 Svært sterke hallusinasjoner

7 Vedvarende hallusinasjoner

______________________________________________

**HODEPINE, TRYKK I HODET**

*Spør: "Føles hodet annerledes? Føles det som om det er et bånd*

*rundt hodet ditt?" Ikke skår for svimmelhet eller ørhet. Forøvrig*

*skåres alvorlighetsgrad.*

0 Ingen

1 Svært lett

2 Lett

3 Moderat

4 Moderat sterk

5 Sterkt

6 Svært sterkt

7 Ekstremt sterk

______________________________________________

**ORIENTERING OG BEVISSTHETSNIVÅ**

*Spør: "Hvilken dag er det i dag? Hvor er du? Hvem er jeg?"*

*Vennligst legg sammen følgende tall…"*

0 Orientert og kan legge sammen flere tall

1 Kan ikke legge sammen flere tall eller er usikker på

dato

2 Ikke orientert for dato, avviker med inntil 2 dager

3 Ikke orientert for dato, avviker med mer enn 2 dager

4 Ikke orientert for sted og/eller person

______________________________________________

Oversatt av Jan Hammer, Trude Bjørnstad, Odd Skinnemoen, Vestre Viken

og Jan Tore Daltveit, Svein Skjøtskift, Thomas Mildestvedt, Haukeland Universitetssjukehus. mai 2014.

# Appendix C – Placebo decleration list

| **Syntocinon/Placebo Nasal Spray #2** | |  |
| --- | --- | --- |
| **5ml** |  |  |
|  |  |  |
| Chlorobutanol, NF (Anhydrous) | 0,013 | gm |
| Methylparaben, NF | 0,002 | gm |
| Propylparaben, NF | 0,001 | gm |
| Citric Acid, USP (Anhydrous) | 0,014 | gm |
| Sodium Chloride, USP (Granular) | 0,025 | gm |
| Sodium Phosphate Dibasic, USP | 0,01 | gm |
| Glycerin, USP | 0,25 | ml |
| QS |  |  |
| Purified Water, USP | 5 | ml |

# Appendix D – example of labelling

|  | |
| --- | --- |
| **Oxytocin/placebo nesespray til klinisk utprøving**  **for hjemmebruk**  (Batchnr, produksjonsdato, legemiddelform, etc) | |
|  | |
| *Protokollnr:* | 140682 |
| *EudraCTno:* | 2015-004463-37 |
| *Ved spørsmål, kontakt:* | Katrine Melby |
|  | Tlf 92886639  [katrine.melby@ladebs.no](mailto:katrine.melby@ladebs.no)  Lade Allé 86, 7041 Trondheim |
| *Åpnet dato:* | Åpent felt til å skrive i |
| *Studiespesifikk pasientkode:* | Åpent felt til å skrive i |
| For hjemmebruk: **1 spray i hvert nesebor inntil 3 ganger daglig**.  Utløpsdato:Åpent felt til å skrive i  **Anvendes senest 6 mndr etter produksjonsdato. Uåpnet pakning skal oppbevares kaldt.**  **Holdbarhet: 1 mnd etter åpning. Oppbevares i romtemperatur.** | |
| **Oppbevares utilgjengelig for barn.** | |

|  | |
| --- | --- |
| **Oxytocin/placebo nesespray til klinisk utprøving**  **ved Lade Behandlingssenter**  (Batchnr, produksjonsdato, legemiddelform, etc) | |
|  | |
| *Protokollnr:* | 140682 |
| *EudraCTno:* | 2015-004463-37 |
| *Ved spørsmål, kontakt:* | Katrine Melby |
|  | Tlf 92886639  [katrine.melby@ladebs.no](mailto:katrine.melby@ladebs.no)  Lade Allé 86, 7041 Trondheim |
| *Åpnet dato:* | Åpent felt til å skrive i |
| *Studiespesifikk pasientkode:* | Åpent felt til å skrive i |
| For bruk i instutisjon: **3 sprayer i hvert nesebor kl 09.00 og kl 18.00**.  Utløpsdato: Åpent felt til å skrive i  **Anvendes senest 6 mndr etter produksjonsdato. Uåpnet pakning skal oppbevares kaldt.**  **Holdbarhet: 1 mnd etter åpning. Oppbevares i romtemperatur.** | |
| **Oppbevares utilgjengelig for barn.** | |
