# Supplementary material for: Actigraphy assessment of motor activity and sleep in patients with alcohol withdrawal syndrome and the effects of intranasal oxytocin
Source: PLoS One. 2020 Feb 13;15(2):e0228700. doi: 10.1371/journal.pone.0228700 (PMC7018062; doi:10.1371/journal.pone.0228700)
Supplement: Attachment — Submitted filename: Response to reviewers.docx [file pone.0228700.s007.docx]

Editor-in-chief

PLOS ONE

Trondheim, Norway, December 2^nd^, 2019

**PONE-D-19-23454 resubmission: «Actigraphy assessment of motor activity and sleep in patients with alcohol withdrawal syndrome and the effects of intranasal oxytocin»**

Dear Editor-in-chief,

We are pleased to resubmit for publication the revised version of our manuscript “Actigraphy assessment of motor activity and sleep in patients with alcohol withdrawal syndrome and the effects of intranasal oxytocin». We appreciate the comments from the two reviewers and the Senior Managing Editor. We have addressed each of the comments as outlined below, with our own response in italics. We have also uploaded one manuscript file where changes are highlighted in yellow, and one without highlighted changes.

We have done our best to adhere to your formatting checklist, including file naming. We have removed the phrase “data not shown”, and all data described are now either presented in detail in the manuscript or available as Supplementary tables.

The full name of the Ethics Committee can be found in the manuscript and in the submission form.

Regarding the availability of individual data from this study. We have now uploaded all data in figshare.com. The DOI will be available after published manuscript. The reason we wanted to make data available upon request is that even if data is anonymized, it still contains potentially identifying or sensitive patient information making the patient recognizable to himself/herself, co-patients at the ward, etc., given the small sample size of the trial, the specific time period the study was carried out, and the relatively small total population of Trøndelag county, Norway.

No results from this manuscript has been published or is under consideration for publication elsewhere. The research protocol was presented as a poster at the The ASAM 48th Annual Conference, April 6-10, 2017, New Orleans, LA, USA and at the 30th ECNP Congress, September 2-5, 2017, Paris, France.

Some other results from this RCT was previously published in Drug and Alcohol Dependence, DOI: 10.1016/j.drugalcdep.2019.01.003. However, this publication did not refer to actigraphy measures, motor activity or sleep, but merely describes the clinical and pharmacological effects of oxytocin in relation to placebo. Actigraphy is a separate field of research, we have included a control group, the research group consists of other people, etc. The results from the current study are therefore best presented as an independent paper.

There are no changes related to the financial disclosure since the first submission.

**Reviewer #1 – comments:**

1 What is the rationale for expecting OT to affect actigraphy and sleep related outcome measures?

*To clarify this issue, changes have been made in Introduction, please cf. the first three paragraphs, which are rewritten.*

2 A more rational approach would be for Part 1 to establish group differences (AUD vs controls) in actigraphy/ sleep outcomes. Then look for an effect of OT on these outcomes in AUD patients in withdrawal with a rationale to do so. That is, there any evidence that OT affects motor activity or sleep (there is some literature on this).

*We discussed this issue thoroughly before we submitted the first version of the paper, and we have now again considered the possibility of changing the order of the two parts. The reason why we did compare the two treatment groups first, was that the origin of this study was a double-blind RCT where two treatment groups, OT and placebo, were compared. Since no statistically significant differences were found between the groups in any of the outcomes (information that we had to present first, i.e. in Part 1), we could view the patient group as one homogenous group, thereby comparing all patients to healthy controls (Part 2). Doing it the other way around would in our opinion be less logical, because the reader would then not know whether it could be justified or not to compare the whole patient group with the healthy controls, i.e. whether the differences would be caused by the underlying condition or the drug treatment.*

3 This seems a lost opportunity to report whether this study replicated a previous study on the effect of OT on alcohol withdrawal symptoms, craving and benzo requirement during withdrawal (Pedersen et al. 2013). If it does not have an effect on withdrawal symptoms then why should it have an effect on activity and sleep disturbances related to alcohol withdrawal.

*We realize that we have not been able to clearly convey the rationale behind this study. The pilot study of Pedersen et al. from 2013 showed a remarkable effect in favour of oxytocin in alcohol withdrawal. Such an effect was not seen in our study where we tried to replicate these results. The clinical results from our study are already published (ref. 19 in the present article). We have now clarified this issue in paragraph 3 in the Introduction as well as in the very first sentence in the Materials and methods section. In the current study, we wanted to explore further whether actigraphy could give a more precise description of the degree of withdrawal symptoms. We also wanted to see whether actigraphy could give more information about the effects of oxytocin, effects that we were not able to reveal in the first study, as we had the idea that actigraphy provides a more accurate and objective measure to motor activity and sleep, than self-reported symptoms do. We have made numerous changes in the manuscript to better describe this rationale.*

**Reviewer #2 – comments:**

- Inclusion and exclusion criteria. There was no information on the inclusion and exclusion criteria for the controls. What are the characteristics of these subjects i.e. medical history, social history, occupation, psychological (stress, anxiety etc) etc. More information to be provided on type of occupation.
  - *The inclusion and exclusion criteria are now described. Moreover, table 1 now contains all relevant information available for the control group as well as the patients.*
- Table 1 requires cosmetic changes. At least one decimal point for the percentages. The symbol % for individual figures to be omitted since it was stated on the left column. There was no alcohol level measurement for the healthy controls. The p value - for the number of subjects to be omitted.
  - *Table 1 is revised as suggested.*
- There was no consideration of the attrition rates in the sample size calculation to derive the sample size.
  - *The power calculation is now included in the Materials and methods section. Attrition rates can now be calculated based on the numbers given (32 patients requested, 40 included). In the Discussion section, we comment on the relevance of the power calculation performed in relation to the present study with focus on actigraphy data.*

Results

- Table 2, decimal points to be standardized. For standardization, the word mean to be written in all variables.
  - *The numbers of decimals are now standardized in all Tables. The word “mean” is included where appropriate; we have in addition included in the legend to the table that all numbers are means ± SDs.*
- Table 3, 2 subjects who were not included in the analysis to be denoted in the table footnote. For the title, n=38 to be stated rather than 40. Please ensure all the numbers stated in the figures and tables title are correct.
  - *The description of the numbers of patients are corrected as suggested.*
- Table 4 and Table 5, results to be presented according to oxytocin group, placebo group and overall group.
  - *For the information presented in Table 4, group-specific data are presented in S1 Table, panel A (placebo) and B (oxytocin). For the information presented in Table 5, group-specific data are presented in S2 Table, panel A (placebo) and B (oxytocin).*
- Table 6, separate group (oxytocin and placebo group) to be presented.
  - *All separate group data are now included in Table 2*.

We appreciate the thorough assessment and comments of our manuscript from the reviewers and the Senior Managing Editor*,* and hope that you will find our revision satisfactorily.

We look forward to hearing from you again.

Please address all correspondence to:

Katrine Melby, MD

Department of Clinical Pharmacology,

St. Olav University Hospital

P.O. Box 3250,

N-7006 Trondheim, Norway

Phone: + 47 92 88 66 39/ Fax: + 47 72 82 91 12

E-mail: [katrine.melby@stolav.no](mailto:katrine.melby@stolav.no)

Yours sincerely,

On behalf of the authors,

Katrine Melby
