# Supplementary material for: Actigraphy assessment of motor activity and sleep in patients with alcohol withdrawal syndrome and the effects of intranasal oxytocin
Source: PLoS One. 2020 Feb 13;15(2):e0228700. doi: 10.1371/journal.pone.0228700 (PMC7018062; doi:10.1371/journal.pone.0228700)
Supplement: Attachment — Submitted filename: Response to reviewers.docx [file pone.0228700.s008.docx]

Editor-in-chief

PLOS ONE

Trondheim, Norway, January 16^th^, 2020

**PONE-D-19-23454R1 resubmission: «Actigraphy assessment of motor activity and sleep in patients with alcohol withdrawal syndrome and the effects of intranasal oxytocin»**

Dear Editor-in-chief,

We are pleased to resubmit for publication the second revised version of our manuscript “Actigraphy assessment of motor activity and sleep in patients with alcohol withdrawal syndrome and the effects of intranasal oxytocin». We appreciate the minor comment from the reviewer. We have addressed the comment as outlined below and changed the revised manuscript accordingly. We have also uploaded one manuscript file where changes are made with ***track changes***, and one without changes.

There are no changes related to the financial disclosure since the first submission.

**Comment from Reviewer #2:**

Authors have put in great effort to address the comments.

Minor comment(s):

Line 175, the symbol > and < for the power and alpha to be replaced with symbol = and the sentence to be rephrased.

**Reply:**

We once again appreciate the thorough assessment of our manuscript from the reviewers and the Senior Managing Editor. We have rewritten the part describing the power analysis as suggested be the reviewer.

We hope that you will find our revision satisfactorily and look forward to hearing from you again.

Please address all correspondence to:

Katrine Melby, MD

Department of Clinical Pharmacology,

St. Olav University Hospital

P.O. Box 3250,

N-7006 Trondheim, Norway

Phone: + 47 92 88 66 39/ Fax: + 47 72 82 91 12

E-mail: [katrine.melby@stolav.no](mailto:katrine.melby@stolav.no)

Yours sincerely,

On behalf of the authors,

Katrine Melby
